# Membrane phospholipid peroxidation promotes loss of dopaminergic neurons in psychological stress‐induced Parkinson's disease susceptibility

**DOI:** 10.1111/acel.13970

**Published:** 2023-08-25

**Authors:** Xiao‐Min Lin, Ming‐Hai Pan, Jie Sun, Meng Wang, Zi‐Han Huang, Guan Wang, Rong Wang, Hai‐Biao Gong, Rui‐Ting Huang, Feng Huang, Wan‐Yang Sun, Hai‐Zhi Liu, Hiroshi Kurihara, Yi‐Fang Li, Wen‐Jun Duan, Rong‐Rong He

**Affiliations:** ^1^ Guangdong Engineering Research Center of Chinese Medicine & Disease Susceptibility/International Cooperative Laboratory of Traditional Chinese Medicine Modernization and Innovative Drug Development of Chinese Ministry of Education (MOE)/Guangdong Province Key Laboratory of Pharmacodynamic Constituents of TCM and New Drugs Research/The First Affiliated Hospital of Jinan University Jinan University Guangzhou China; ^2^ Innovation Center of Nursing Research, Nursing Key Laboratory of Sichuan Province, State Key Laboratory of Biotherapy and Cancer Center, West China Hospital, Collaborative Innovation Center of Biotherapy Sichuan University Chengdu China; ^3^ School of Chinese Materia Medica and Yunnan Key Laboratory of Southern Medicinal Utilization Yunnan University of Chinese Medicine Kunming China; ^4^ State Key Laboratory of Quality Research in Chinese Medicine Macau University of Science and Technology Macau China

**Keywords:** chronic stress, ferroptosis, leonurine, membrane lipid peroxides, Parkinson's disease

## Abstract

Parkinson's disease (PD) is a neurodegenerative disorder associated with *α*‐synuclein aggregation and dopaminergic neuron loss in the midbrain. There is evidence that psychological stress promotes PD progression by enhancing glucocorticoids‐related oxidative damage, however, the mechanisms involved are unknown. The present study demonstrated that plasma membrane phospholipid peroxides, as determined by phospholipidomics, triggered ferroptosis in dopaminergic neurons, which in turn contributed to stress exacerbated PD‐like motor disorder in mice overexpressing mutant human *α*‐synuclein. Using hormonomics, we identified that stress stimulated corticosteroid release and promoted 15‐lipoxygenase‐1 (ALOX15)‐mediated phospholipid peroxidation. ALOX15 was upregulated by *α*‐synuclein overexpression and acted as a fundamental risk factor in the development of chronic stress‐induced parkinsonism and neurodegeneration. Further, we demonstrated the mechanism by which corticosteroids activated the PKC pathway and induced phosphatidylethanolamine‐binding protein‐1 (PEBP1) to form a complex with ALOX15, thereby facilitating ALOX15 to locate on the plasma membrane phospholipids. A natural product isolated from herbs, leonurine, was screened with activities of inhibiting the ALOX15/PEBP1 interaction and thereby attenuating membrane phospholipid peroxidation. Collectively, our findings demonstrate that stress increases the susceptibility of PD by driving membrane lipid peroxidation of dopaminergic neurons and suggest the ALOX15/PEBP1 complex as a potential intervention target.

## Graphical abstract

Chronic stress accelerates ALOX15‐mediated accumulation of phospholipid hydroperoxides through the signalling of the corticosteroid. Corticosterone activates the PKC pathway that dissociates PEBP1 from Raf‐1 to bind ALOX15, which assembles into a complex that catalyzes the phospholipid peroxidation. Leonurine attenuates phospholipid peroxidation by preventing the assembly of the ALOX15/PEBP1 complex, thereby alleviating chronic stress‐induced Parkinson's disease susceptibility.

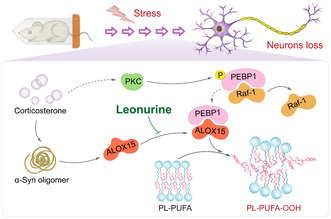

**Abbreviations**
4‐HNE: 4‐hydroxynonenal
A53T: transgenic mice overexpressing mutant human α‐synuclein
AA: arachidonic acid
ACSL4: Acyl‐CoA synthetase long‐chain family member 4
ALOX15: 15‐lipoxygenase‐1
CETSA: cell thermal shift assay
CORT: corticosterone
Fer‐1: ferrostatin‐1
GPX4: glutathione peroxidase 4
GSH: glutathione
HPA: hypothalamic‐pituitary‐adrenal
LA: linoleic acid
Leo: Leonurine
LPCAT3: lysophosphatidylcholine acyltransferase 3
MDA: Malondialdehyde
MPTP: 1‐methyl‐4‐phenyl‐1,2,3,6‐tetrahydropyridine
MST: microscale thermophoresis assay
NDGA: Nordihydroguaiaretic acid
ORAC: Oxygen radical absorbance capacity
PD: Parkinson's disease
PE: phosphatidylethanolamine
PEBP1: phosphatidylethanolamine‐binding protein‐1
PTGS2: prostaglandin‐endoperoxide synthase 2
STS: staurosporine
TG: Tianma Gouteng granule
WT: wild‐type

## 1 INTRODUCTION

Parkinson's disease (PD) is a progressive neurodegenerative disorder characterized by the degeneration of dopaminergic neurons as well as Lewy bodies formed by *α*‐synuclein oligomers in the substantia nigra, also known as synucleinopathies (Kalia & Lang, 2015). In the development of PD, various factors have been implicated, including exposure to pesticides (Pezzoli & Cereda, 2013), head injuries (Taylor et al., 2016) and psychological stress (Rod et al., 2010). As a state similar to sub‐health, psychological stress is manifested by impaired physiological functions, decreased nonspecific resistance, which increase susceptibility to disease (Pan et al., 2020). Those who suffer from chronic psychological stress, such as depressive emotions, are more susceptible to develop neurodegenerative diseases including PD. The available evidence suggests that psychological stress contributes to the incidence of dyskinesia (Sorenson et al., 2013), cognitive decline (Pesonen et al., 2013), and dysfunction (Kulmala et al., 2013) in non‐PD patients, as well as to the development of disease in PD patients (Ibrahimagic et al., 2016). Additionally, chronic restraint‐induced depressive‐like stress accelerated the onset of parkinsonism in both wild‐type (WT) mice treated with 1‐methyl‐4‐phenyl‐1,2,3,6‐tetrahydropyridine (MPTP) (Janakiraman et al., 2017) and transgenic mice overexpressing mutant human *α*‐synuclein (encoded by *SNCA* ^A53T^, referred to as A53T) (Zhang et al., 2018). The exact mechanism of stress influence, however, remains unclear. An adaptation to stress is mediated by the hypothalamic–pituitary–adrenal (HPA) axis that releases corticosteroids. Corticosteroid levels above normal have been linked with oxidative stress and decreased glutathione (GSH), which are two of the main causes of neuronal destruction (Camargo et al., 2018; Jin et al., 2019).

An expanding body of research relates corticosteroids to the signaling pathway in ferroptosis, a new form of regulated cell death that is triggered by membrane lipid peroxides, specifically oxidized phosphatidylethanolamine species (oxPEs) (Dixon et al., 2012; Kagan et al., 2017). In osteoporosis caused by corticosteroids, ferroptosis‐associated proteins, prostaglandin‐endoperoxide synthase 2 (PTGS2), and Acyl‐CoA synthetase long‐chain family member 4 (ACSL4) levels were upregulated, while glutathione peroxidase 4 (GPX4) levels were decreased. In addition, ACSL4 and lysophosphatidylcholine acyltransferase 3 (LPCAT3) were found to be highly expressed in the adrenal cortex, which produces corticosteroids, whereas inhibition of steroid hormone secretion reversed RSL3‐induced ferroptosis (Yang et al., 2021). In our latest study, ferroptosis has been shown to contribute to the loss of dopaminergic neurons in PD (Sun et al., 2023). In this regard, it may be possible to link the susceptibility of stress‐induced PD to the presence of lipid peroxidation‐related ferroptosis in dopaminergic neurons. It was revealed in this study that chronic restraint stress increased corticosteroid levels, which consequently caused dopaminergic neurons to undergo ferroptosis as a result of phospholipid peroxidation, thus increasing the susceptibility to PD. Furthermore, leonurine has been identified as a neuroprotective compound against ferroptosis by inhibiting phospholipid peroxidation.

## 2 METHODS

### 2.1 Animals

*SNCA* ^A53T^ mice (stock No. #006823) and *Alox15* ^−/−^ mice (stock No. #002778) were obtained from Jackson Laboratory (Bar Harbor, ME, USA). Age‐matched WT littermates were used as control mice and all mice were of the C57BL/6J background. The mice were genotyped by PCR of DNA extracted from a tail biopsy and littermates were always used as controls. All animals were group housed in a room at a mean constant temperature (25 ± 2°C), humidity (55 ± 5%) and illumination (12 h light/dark cycle), and free access to standard pellet chow and water in the Medical Experimental Animal Center of Jinan University. All animal experiments are conducted in accordance with animal humane care standards. All animal care and experimental procedures have been approved by the Experimental Animal Ethics Committee of Jinan University. All animal treatments follow the National Institutes of Health's Guide for the Care and Use of Laboratory Animals (NIH publication No. 80‐23, revised in 1996) and were approved by the Animal Ethics Committee of Jinan University (Approval No.: 20130904001).

#### 2.1.1 Chronic restraint stress in mice

Four weeks were spent subjecting mice in the stress group to a 4‐h restraint stress load from 17:00 to 21:00 for six consecutive days per week. Behavioral tests were performed 1 day after the stress. For the purpose of control, non‐stressed mice also experienced water, and food deprivation during the restraint period.

#### 2.1.2 Corticosterone (CORT) treatment in mice

Four weeks were spent subjecting mice in the CORT group to a subcutaneous injection of 2 mg/kg (dissolved in 0.1 mL polyethylene glycol) for six consecutive days per week. Behavioral tests were performed 1 day after the stress. The same volume of polyethylene glycol was injected into control mice.

### 2.2 Pole test

The purpose of the pole test was to record the coordination ability of the mice. The pole was composed of an iron frame with a diameter of 1 cm and a height of 60 cm and a 1.5 cm diameter ball fixed on the top of it. Gauze was wrapped around the surface of the ball to prevent the mice from slipping off. The mice were trained a week in advance to adapt. During the experiment, the mice were placed on the pole with their heads up, mice climbed to the bottom pole time was recorded. If the time exceeded 60 s, the time was measured by 60 s. The test was repeated for five times to obtain the average value. To avoid interference, the experiment was carried out in a quiet environment.

### 2.3 Rotarod test

In the rotarod test, the coordination ability of mice was detected by recording the retention time of mice on the cylindrical rotarod. The mice were trained to stay on the rod of the rotarod (Ugo Basile S.R.L., Gemonio VA, Italy) 3 days prior to the actual test and the speed was set to evenly accelerate from 5 to 30 rpm in 300 s. After training, the rod speed was increased from 5 to 30 r/min within 300 s until the mouse fell from the rod within the measurement time limit. The test interval of each trail was at least 30 min break, and each mouse was repeatedly measured three times at the average speed. All tests and measurements were performed on the same day. Experiments were conducted in a quiet environment to avoid interference.

### 2.4 Gait analysis

Gait analysis is a system of rodent gait analysis, including balance maintaining ability, limb use, motion state, and gait stability analysis by using the CatWalk™ XT (Noldus Information Technology, Wageningen, The Netherlands). Before the formal test, the mice should be trained three times. After the experiment, Noldus CatWalk XT 9.1 system was used for statistical analysis of the data obtained. The experiment process is as follows: mice passed through the glass corridor, and the fluorescent lights illuminated the corridor, allowing the charge coupled device (CCD) camera below to capture images of the footprint. Repeat the actual test until five consecutive uninterrupted runs were recorded. All tests and measurements were performed on the same day. Experiments were conducted in a quiet environment to avoid interference.

### 2.5 Brain stereotactic injection

Animals were anaesthetized with 1.5% isoflurane mixed with medical air (20% oxygen, 80% nitrogen) at a flow rate of 0.7 L/min and mounted in a stereotaxic apparatus fixed with a mouse adaptor. The lentivirus was injected into the substantia nigra at a flow rate of 0.1 μL/min with a final volume of 0.5 μL, according to the following coordinates: anteroposterior (AP) = 3.08 mm, mediolateral (ML) = −1.5 mm and dorsoventral (DV) = −4.5 mm. The needle was left in the injection site for 5 min to avoid reflux. After surgery, mice were allowed to recover for 7 days after a single injection before any other treatment. Details of the AAVs used are shown in Table S3.

### 2.6 Immunohistochemistry

GTVisionTM Anti‐Mouse/Rabbit Universal Immunohistochemistry Kit (GenTech Biotechnology, Shanghai, China) was applied for observation of mice dopaminergic neurons. After anesthesia with 4% chloral hydrate, the heart was perfused with physiological saline, and then with 4% paraformaldehyde in 0.1 M phosphate buffer (pH 7.4) to obtain brain tissue. Whole brains were immersed in 4% paraformaldehyde at 4°C for 1 week, embedded in paraffin and cut into 5 μm sections. After PBS washing and the antigen retrieval, the sections were blocked with 10% 0.1 M normal goat serum and incubated with mouse anti‐TH primary antibody overnight at 4°C, followed by secondary antibody for 2 h at room temperature. The sections were stained with DAB and stained with nuclei in hematoxylin. Images were captured with a microscope (Olympus, Tokyo, Japan).

### 2.7 Malondialdehyde (MDA) measurement

The MDA content in tissues was determined with Lipid Peroxidation MDA Assay Kit (Beyotime Technology, Shanghai, China, S0131), according to the protocol provided by the manufacturer's instructions.

### 2.8 Determination of glutathione (GSH) by HPLC‐ECD analysis

The tissue was separated on ice and an equal volume of 6% perchloric acid was added before homogenization. The tissue suspension was then centrifuged at 10000 × *g* for 15 min at 4°C, and the supernatants were transferred into HPLC vials for GSH analysis. An Agilent Eclipse Plus C18 reverse phase column (HSS T3 1.8 μm, 100 × 2.1 mm, Waters Acquity) was used for the analysis, and the mobile phase ratio was methanol: water = 10:90. The parameters were set to a column temperature of 33°C, a detection voltage of 0.52 V, and a flow rate of 1 mL/min. The contents of GSH, dopamine, DOPAC, and HVA were calculated according to the standard curve. Standards (Millipore Sigma) were accurately weighed and diluted to a series of concentrations of 5, 10, 20, 50, 100, 200, 400, 600, and 800 μg/L using a mobile phase solution.

### 2.9 LC–MS/MS‐based phospholipidomics analysis

Phospholipids were extracted by the reported Folch method and were further analyzed by LC–MS using normal‐phase (silica) chromatography system coupled with Q‐Exactive Hybrid Quadrupole‐Orbitrap mass spectrometer (Thermo Fisher Scientific). Phospholipids were separated by a Dionex UltiMate 3000 DGLC standard system (Thermo Fisher Scientific) at a flow rate of 0.2 mL/min on normal‐phase column (Phenomenex Luna Silica, 3 μm, 150 × 2.0 mm, Danaher Corporation, Washington, DC, USA). The column temperature was maintained at 35°C. The mobile phase consisted of 10 mM ammonium formate in propanol/hexane/water (285:215:5, v/v/v, solvent A) and 10 mM ammonium formate in propanol/hexane/water (285:215:40, v/v/v, solvent B). The linear gradient conditions were as follows: 0 min, 10% B; 20 min, 32% B; 30 min, 70% B; 32 min, 100% B; 58 min, 100% B; 60 min, 10% B; 75 min, 10% B. The injection volume was 2 μL. MS/MS analysis of phospholipids was performed on a Q‐Exactive Hybrid Quadrupole‐Orbitrap mass spectrometer (Thermo Fisher Scientific). Analysis was performed in full MS negative mode at resolution setting of 70,000 and data‐dependent‐MS/MS mode at resolution setting of 17,500. For MS, the scan range was 400–1800 *m/z* and the maximum ion injection time was 600 ms using 1 microscan per MS scan. For MS/MS, high energy collision induced dissociation (HCD) analysis was performed with the collision energy set to 24 eV and the maximum ion injection time of 200 ms. The inclusion list included all species of phospholipids and their oxidized/deuterated products. An isolation window of 1.0 Da was set for the MS and MS/MS scans. Capillary spray voltage was 3.0 kV, and capillary temperature was 320°C. The S‐lens Rf level was 60.

### 2.10 LC–MS analysis of dopamine

Dopamine was determined by LC–MS using a Triple Quad™ 6500 Mass Spectrometer (SCIEX). Analytes were separated on a C18 column (Acquity HSS T3, 1.8 μm, 2.1 mm × 100 mm, 40°C, Waters) at a flow rate of 0.4 mL/min on an Exion LC AD system. Gradient: solvent A (water) and solvent B (acetonitrile), each containing 0.1% formic acid (v/v): 0–1.2 min isocratic of 2% B, 1.2–2.5 min linear gradient of 2%–60% B, 2.5–4.5 min linear gradient of 60%–95% B, 4.5–5.0 min isocratic of 95% B, and 5.0–8.5 min re‐equilibration of 2% B. Positive and negative ion modes switching with a scheduled MRM scan method was used. Capillary spray voltage: 5.5 and −4.5 kV; ion source temperature, 600°C. Nebulizer gas and heater gas were set to 55 psi, and curtain gas was set to 30 abr. Data acquisition and processing were carried out using Analyst 1.6.2 software (SCIEX).

### 2.11 Cell line and culture

The *SNCA* ^A53T^ PC12 conditional overexpression cell line was a gift from the Chinese Academy of Sciences and cultured in Dulbecco's modified Eagle's medium (DMEM) supplemented with 10% horse serum (HBS, Gibco) and 5% fetal bovine serum (FBS, Gibco) at 37°C with 5% CO_2_ humidified atmosphere. The overexpression of *SNCA* ^A53T^ was induced by doxycycline hydrochloride (1 μg/mL) for 24 h (abbreviated as iA53T).

### 2.12 Assessment of cell viability

Cells were dispensed in 96‐well plate at a density of 7 × 10^3^ cells per well. After 24‐h incubation, cells were treated with the tested reagents for the indicated periods of time and treat with Cell Counting Kit‐8 (TargetMol, USA). Optical density was measured using a multimode reader.

### 2.13 Western blot analysis

Tissues or cell samples were lysed in a lysis buffer supplemented with a protease inhibitor cocktail and the supernatants were collected after centrifugation at 13,000 × *g* for 15 min. Protein samples were quantified using Pierce BCA protein assay kit. Samples were desaturated (10 min, 100°C) with 5 × loading buffer. Afterwards, protein samples (30 μg per well) were separated in 8%–15% gradient SDS‐PAGE and transferred to PVDF membrane (MilliporeSigma, Burlington, MA, USA). Membranes were incubated at 4°C overnight in the presence of primary antibodies: anti‐GPX4 (ab125066), anti‐4‐HNE (ab46545), anti‐TH (ab112), anti‐DAT (ab184451), anti‐*α*‐synuclein (sc‐7011‐R), anti‐PEBP1 (sc‐376925), anti‐ERK1/2 (11257‐1‐AP), anti‐*p*‐ERK1/2 (28733‐1‐AP), anti‐*p*‐PEBP1 (sc‐135779), anti‐TFRC (10084‐2‐AP), anti‐GAPDH (FD0063), anti‐iPLA_2_ *β* (sc‐376563), anti‐c‐Raf (53745S), anti‐HA (51064‐2‐AP), anti‐Flag (F1804), or anti‐GFP (50430‐2‐AP). Membranes were incubated with HRP‐conjugated secondary antibodies at room temperature: anti‐rabbit IgG (FDR007) or anti‐mouse IgG (FDM007). The chemiluminescence signals were detected with Pierce® ECL Western blotting Substrate (Thermo Fisher Scientific) as the substrate of HRP on imaging system (Tanon Science & Technology, Shanghai, China) in a dark chamber. Integrated gray values of each band were measured using ImageJ (National Institutes of Health, Bethesda, USA). Details of the plasmids used are shown in Table S3.

### 2.14 Quantitative reverse transcription polymerase chain reaction (RT‐qPCR)

Total RNA was extracted from mice tissues using TRIzol reagent (TaKaRa, Dalian, China). RNA purity and concentration were determined by optical density measurement at 260 nm on a spectrophotometer (IMPLEN, Germany). Then, RNA was reversely transcribed to cDNA using an iScript cDNA synthesis kit (TIANGEN, Beijing, China). qPCR was performed SYBR Green qPCR Mix (TransGen Biotech) according to the manufacturer's instructions. The results were normalized to GAPDH. Primers are listed in Table S1.

### 2.15 Assessment of lipid peroxidation

Cells were dispensed in dishes at a density of 5 × 10^5^ cells. After treatment, cells were incubated with C_11_‐BODIPY^581/591^ (10 μM) for 30 min. Then the cells collected for analysis in a BD FACSCanto II flow cytometer.

### 2.16 Immunofluorescence

HEK293 cells were dispensed in confocal dishes at a density of 5 × 105 cells. Cells were transfected with both ALOX15‐GFP and PEBP1‐HA plasmids after cell apposition, and CORT was given for 24 h after plasmid transfection. After treatment, cells were fixed in 4% formaldehyde for 15 min at room temperature and then permeabilized with 0.1% Triton X‐100 for 15 min, and blocked with 5% bovine serum albumin in PBS at 37°C for 2 h. The cells were incubated with the primary antibody (HA, 1:100) at 4°C overnight, followed by Alexa Fluor 555‐conjugated secondary antibodies (1:500, Thermo Fisher Scientific, A21424). After additional PBS washes, the cells were counterstained with the nuclear stain 4,6‐diamidino‐2‐phenylindole (C1002, Beyotime) at room temperature for 10 min. A Laser Scanning Confocal Microscope (Olympus SpinSR10) was used to acquire immunofluorescence images.

### 2.17 Oxygen radical absorbance capacity (ORAC)

The antioxidant capacity was measured using ORAC assay (Oxygen Radical Absorbance Capacity) according to the literature (Li et al., 2018). Briefly, fluorescence sodium (Sigma, St. Louis, MO, USA) was used as a fluorescence probe, Trolox as a standard free radical scavenger and AAPH as the peroxyl radical generator. Reaction mixture consists of 20 μL of 75 mM phosphate‐buffered saline (PBS), 20 μL of leonurine (1, 10, 20 μM), 20 μL of fluorescein sodium and 140 μL of AAPH. The reaction was initiated with the addition of AAPH. Fluorescence was measured by a TECAN GENios fluorescence microplate reader (Männedorf, Switzerland) at 37°C with excitation/emission at 485/535 nm and detected every 2 min for 50 min. The ORAC value was calculated as the net area under the fluorescence decay curve using 10 μM of Trolox as the calibration standard.

### 2.18 Microscale thermophoresis (MST) assay

The Monolith NT.115 instrument (NanoTemper, America) was used to determine the binding affinity of the leonurine to ALOX15. The ALOX15 protein labeling was according to the procedure of the Monolith Protein Labeling Kit RED‐NHS 2nd Generation kit. Leonurine at different concentrations was mixed with labeled ALOX15 and incubated for 30 min at room temperature. The intensity of the light‐emitting diode was set to 20%, the excitation power was set to 5%, and the temperature was set to 25°C. The specimens were loaded and measured on the instrument. The data were analyzed using MO. Affinity Analysis v2.3 software (NanoTemper Technologies) (Xiang et al., 2021).

### 2.19 Molecular docking

The x‐ray crystal structure of ALOX15 (PDB ID: 1LOX) was downloaded from the Protein Data Bank (https://www.rcsb.org/). Molecular docking was carried on CDOCKER module of Discovery Studio 2016 (version 3.5; Accelrys, San Diego, CA). The compounds were energy minimized by CHARMm force field. Docking parameters were set according to the standard values.

### 2.20 Statistics analysis

All data were presented as means ± SD of independent experiments. The data were analyzed by IBM SPSS Statistics 26.0 (SPSS Inc., Chicago, IL, USA). *p* values were determined by independent‐samples *t* test, one‐way ANOVA with Bonferroni, Dunnett T3, Tukey HSD and LSD‐post hoc, and two‐way repeated measures. *p* < 0.05 is considered statistically significant.

## 3 RESULTS

### 3.1 PD susceptibility is enhanced by stress‐induced phospholipid peroxidation

**FIGURE 1:**
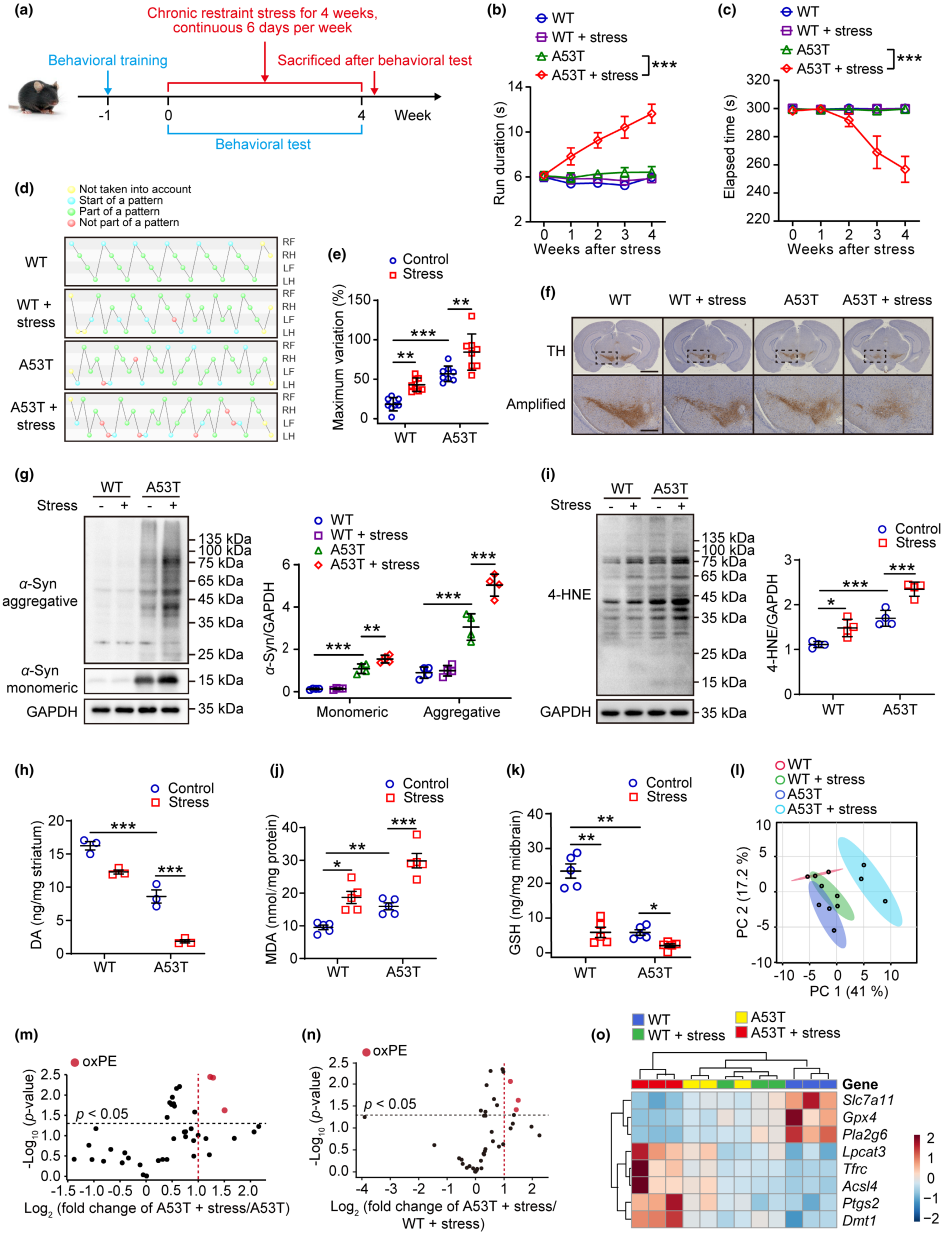
Stress accelerates parkinsonism and phospholipid peroxidation in A53T mice. (a) Time course of the experimental procedure. Mice were initially subjected to the behavioral training for 1 week, and then exposed to restraint stress on four successive experimental weeks. Behavioral tests were conducted and recorded weekly. (b–e) The pole test (b), rotarod test (c), and CatWalk gait analysis (d, e) were used to evaluate the motor function of 9‐month‐old WT and A53T mice after restraint stress. RF, right front; RH, right hind; LF, left front; LH, left hind. Green balls: normal pattern. Red balls: abnormal pattern. Blue balls: the beginning of a walking cycle. Yellow balls: data that were excluded from the test (*n* = 8). (f) IHC of coronal brain sections labeled with tyrosine hydroxylase (TH) antibody and hematoxylin (upper, scale = 2 mm). The substantia nigra (dotted area) were amplified on bottom with scale = 500 μm (*n* = 3). (g) Western blotting (left) and quantitative analysis (right) of *α*‐synuclein (*α*‐Syn) expression in the midbrain (*n* = 4). (h) The dopamine was measured in striatum (*n* = 3). (i) Western blotting (left) and quantitative analysis (right) of 4‐hydroxynonenal (4‐HNE)‐modified proteins in the midbrain (*n* = 4). (j, k) The contents of malondialdehyde (MDA) (*n* = 5) and glutathione (GSH) (*n* = 5) were measured in the midbrain. (l–n) Data of oxidized phospholipids were interpreted by principal component analysis (PCA) and volcano plots. (o) Ferroptosis‐related genes in the midbrain were detected by RT‐qPCR assay and the relative expressions were displayed as heatmap. All data represent mean ± SD. *p < 0.05, **p < 0.01, and ***p < 0.001, by two‐way ANOVA with Tukey (for b, c) and one‐way ANOVA with Tukey (for e), Bonferroni (for g–i) or Dunnett T3 test (for j, k).

Aiming to discover the impact of physiological stress on PD, 9‐month‐old A53T mice were subjected to 4 h of chronic restraint stress per day prior to the onset of the disease (Figure 1a). There was an apparent weight loss in both WT and A53T mice (Figure S1a). As a result of stress, impaired motor coordination (Figure 1b,c) and disordered gait were accelerated in A53T mice in comparison to WT mice (Figure 1d,e; Figure S1b–j). The number of TH‐positive neurons, TH expression in the midbrain and DAT expression in the striatum of the synaptic terminals were significantly decreased (Figure 1f; Figure S1k). In response, DA levels in the striatum were significantly reduced (Figure 1h). Also, A53T mice suffering from stress exhibited higher levels of both expression and high molecular weight species of *α*‐synuclein in the midbrain (Figure 1g). In light of mounting evidence suggesting that membrane lipid peroxides contribute to neurodegeneration (Ko et al., 2021; Sun et al., 2023), we measured the levels of lipid peroxidation end products aldehyde 4‐hydroxynonenal (4‐HNE) and MDA in the midbrain of A53T mice, which were both elevated following stress (Figure 1i,j). In parallel, the level of GSH declined significantly (Figure 1k). Further, phospholipidomics using LC–MS/MS revealed extensive phospholipid peroxidation in the midbrain of stressed A53T mice, with ferroptosis markers, phosphatidylethanolamine peroxides (ox‐PEs), being the most prominent (Figure 1l–n; Figure S1l–n). Aside from that, stress also altered the proteins and genes that are involved in the ferroptotic pathway (Figure 1o; Figure S1o). Taken together, these results suggest that stress may increase PD sensitivity through phospholipid peroxidation.

### 3.2 A stress‐induced rise in CORT contributes to phospholipid peroxidation and PD susceptibility

**FIGURE 2:**
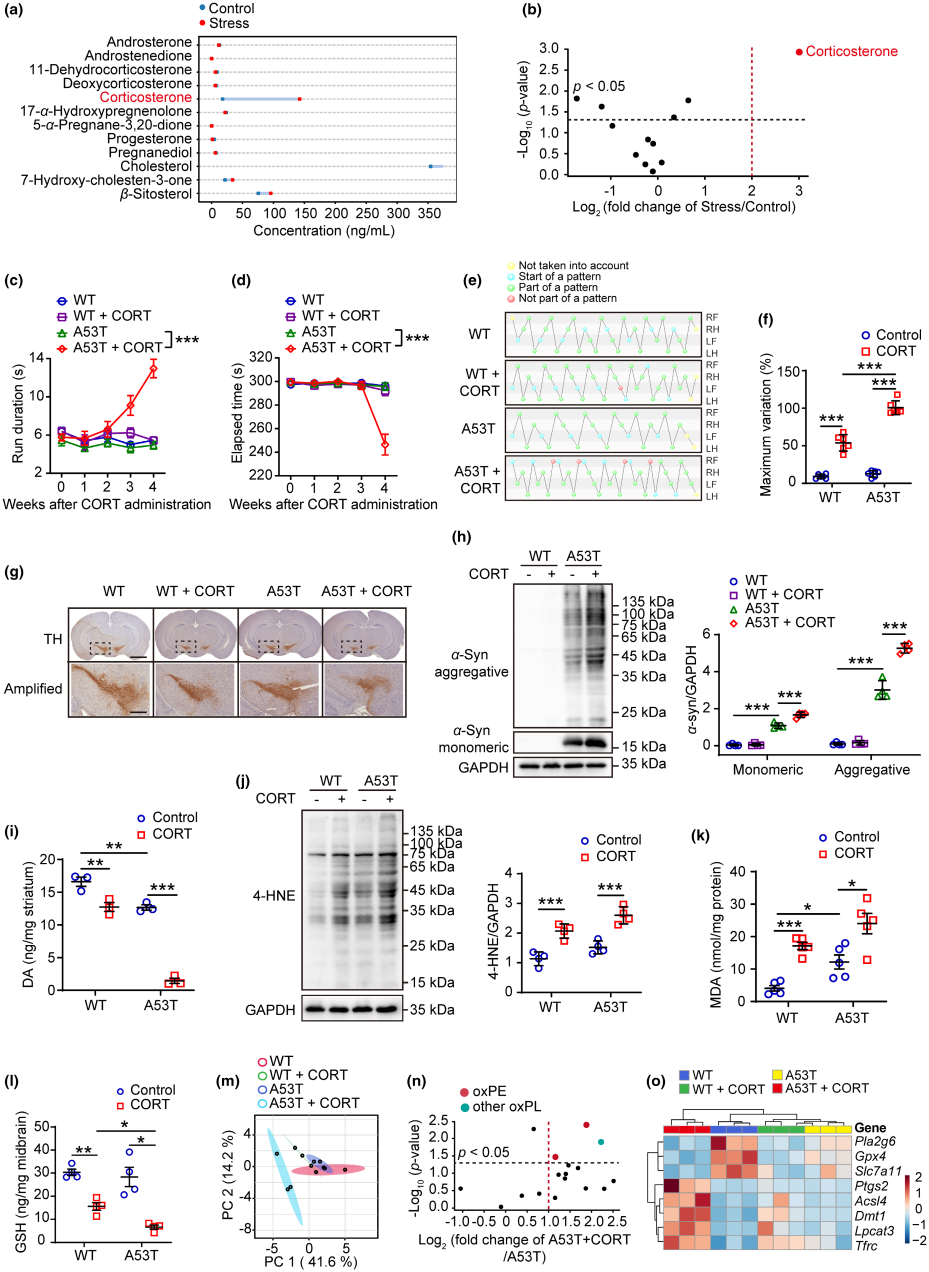
CORT mediates stress‐induced PD susceptibility in A53T mice. (a, b) The dumbbell chart and volcano plot depicted significant changes in plasma hormones concentrations in stressed mice (*n* = 3). (c–f) The pole test (c), rotarod test (d) and Catwalk gait analysis (e, f) were used to evaluate the motor function of 6‐month‐old WT and A53T mice after CORT treatment (2 mg/kg, sc). RF, right front; RH, right hind; LF, left front; LH, left hind. Green balls: normal pattern. Red balls: abnormal pattern. Blue balls: the beginning of a walking cycle. Yellow balls: data that were excluded from the test (*n* = 6). (g) IHC of coronal brain sections labeled with TH antibody and hematoxylin (upper, scale = 2 mm). The substantia nigra (dotted area) were amplified on bottom with scale = 500 μm (*n* = 3). (h) Western blotting (left) and quantitative analysis (right) of *α*‐Syn expression in the midbrain (*n* = 4). (i) The dopamine was measured in striatum (*n* = 3). (j) Western blotting (left) and quantitative analysis (right) of 4‐HNE‐proteins in the midbrain (*n* = 4). (k, l) The contents of MDA (*n* = 5) and GSH (*n* = 4) were measured in the midbrain. (m, n) Data of oxidized phospholipids were interpreted by principal component analysis (PCA) and volcano plots. (o) Ferroptosis‐related genes were in the midbrain detected by RT‐qPCR assay and the relative expressions were displayed as heatmap. All data represent mean ± SD. *p < 0.05, **p < 0.01, and ***p < 0.001, by two‐way ANOVA with Tukey (for c, d) and one‐way ANOVA with Tukey (for f, k), Bonferroni (for h, i) or Dunnett T3 test (for l).

In line with the theory that rodents' central stress response systems, which are controlled by the HPA axis, usually respond adaptively by releasing corticosteroids (Joseph & Whirledge, 2017), the results of hormoneomics analysis revealed that CORT was the most noticeable altered hormone in mice subjected to stress (Figure 2a,b). In A53T mice, CORT exacerbated weight loss (Figure S2a), behavioral disorders (Figure 2c–f; Figure S2b–j), dopaminergic neuron loss (Figure 2g; Figure S2k), reduced DA levels in the striatum of synaptic terminals (Figure 2i) and accumulation of high molecular weight species of *α*‐synuclein (Figure 2h), similar to those caused by stress. It was observed that CORT treatment significantly increased 4‐HNE and MDA, while GSH was significantly decreased in A53T mice (Figure 2j–l). LC–MS/MS analysis of CORT‐treated A53T mice revealed an increase in oxidized phospholipids, including oxPEs, in the midbrain, which is consistent with the effects of stress (Figure 2m,n; Figure S2l–n). Moreover, CORT treatment altered the proteins and genes involved in ferroptosis‐related pathways (Figure 2o; Figure S2o). The following experiments were conducted in PC12 cells overexpressing the human mutation A53T induced by doxycycline (referred as iA53T). Our study showed that PC12 cells overexpressing A53T were more susceptible to ferroptosis inducer RSL3 (Figure S3a). CORT dose‐dependently enhanced A53T‐induced lipid peroxidation as evidenced by the level of 4‐HNE and oxidation of C11‐Bodipy (Figure S3b–d). Additionally, CORT treatments resulted in an increased high molecular weight species of *α*‐synuclein as well as alterations of gene and protein expression related to ferroptosis (Figure S3e–g). These results indicate that CORT may play a major role in promoting phospholipid peroxidation during stress, which in turn contributes to the susceptibility of PD.

### 3.3 The phospholipid peroxidation induced by CORT is mediated by ALOX15 binding to PEBP1

**FIGURE 3:**
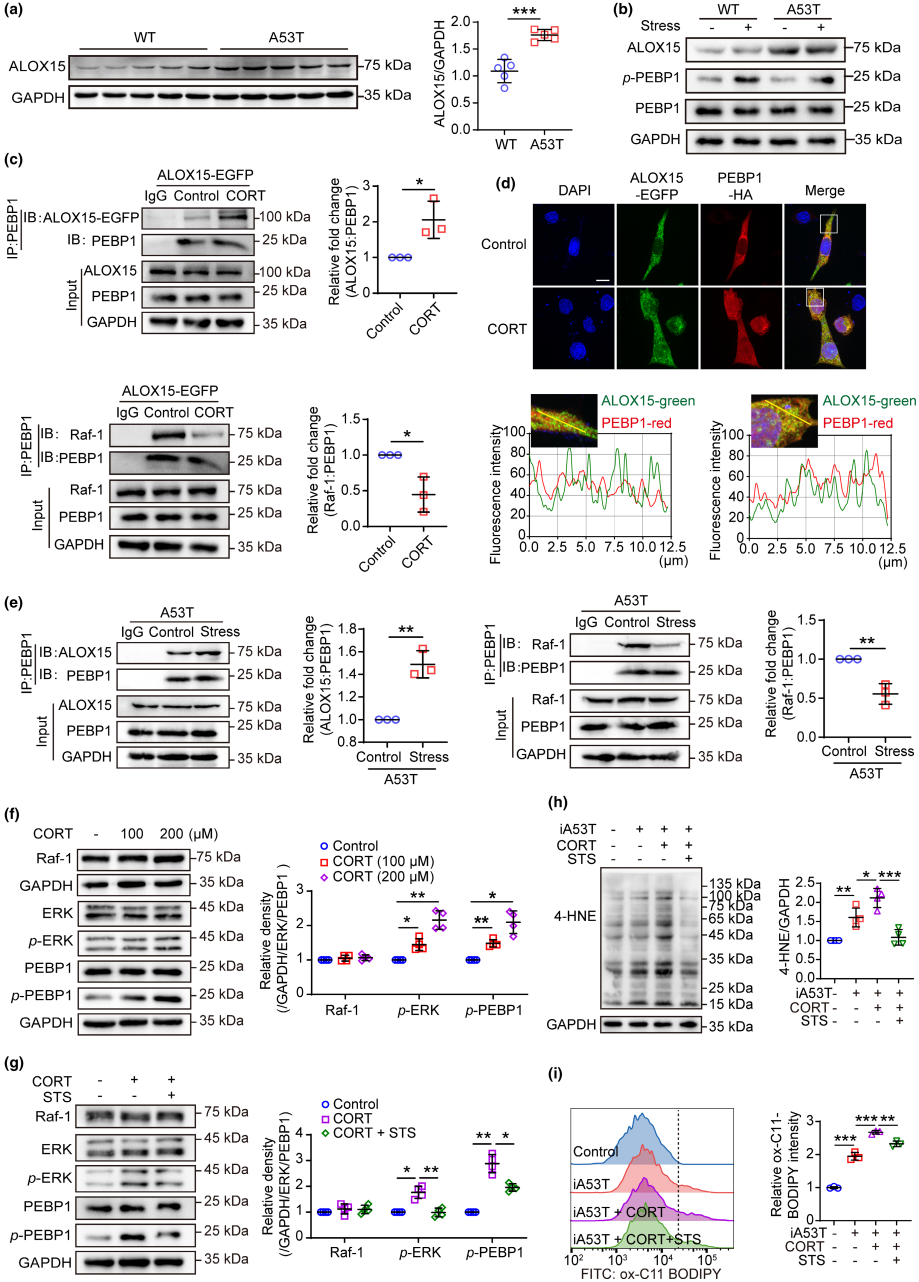
CORT facilitates the interaction of PEBP1 and ALOX15 which increases phospholipid peroxidation. (a) Western blotting (left) and quantitative analysis (right) of ALOX15 expression in the midbrain of WT and A53T mice (*n* = 5). (b) Protein levels of ALOX15, *p*‐PEBP1, and PEBP1 in the midbrain were measured using western blot (*n* = 4). (c) The binding of PEBP1 with either ALOX15 or Raf‐1 that were examined by co‐IP in PC12 cells overexpressing ALOX15‐EGFP after CORT treatment (200 μM) for 24 h (*n* = 3). (d) The co‐localization of ALOX15 with PEBP1 was detected under DMSO or CORT treatment by laser confocal imaging in HEK293 cells overexpressing ALOX15‐EGFP and PEBP1‐HA (scale = 5 μm). The statistics of co‐localization by ImageJ are shown below. (e) The binding of PEBP1 to ALOX15 or Raf‐1 in the untreated or stressed midbrain was detected by co‐IP in A53T mice (*n* = 3). (f, g) Western blotting (left and middle) and quantitative analysis (right) of Raf‐1, *p*‐ERK and *p*‐PEBP1 in PC12 cells treated with indicated agents for 24 h (*n* = 4). CORT, 200 μM. STS, 10 nM. (h) Western blotting (left) and quantitative analysis (right) of 4‐HNE‐protein adducts in *SNCA*‐overexpressed PC12 cells (*n* = 4). CORT, 200 μM. STS, 10 nM. (i) Lipid peroxidation was detected by flow cytometry with C11 BODIPY (581/591) in *SNCA*‐overexpressed PC12 cells (*n* = 3). CORT, 200 μM. STS, 10 nM. All data represent mean ± SD. *p < 0.05, **p < 0.01, and ***p < 0.001, by independent‐samples *t* test (for a, c, e), one‐way ANOVA with Dunnett T3 (for f, i) or Bonferroni (for g, h).

The PE‐binding protein 1 (PEBP1) normally binds to Raf‐1 kinase and inhibits Raf‐1‐activated MEK/ERK pathways (Yeung et al., 1999), whereas PKC phosphorylation would lead to the dissociation of PEBP1 from Raf‐1 and activation of downstream proteins (Trakul & Rosner, 2005). There is evidence that PEBP1 released from Raf‐1 binds 15‐lipoxygenase‐1 (ALOX15) and forms a complex that catalyzes the oxidation of polyunsaturated fatty acid‐containing membrane lipids such as PEs, which in turn initiate ferroptotic cell death (Wenzel et al., 2017). An interesting observation was that A53T mice showed a significant upregulation of ALOX15 expression (Figure 3a), which was irrelevant to stress (Figure 3b). A significant increase in PEBP1 phosphorylation was observed in stressed WT and A53T mice, suggesting that PEBP1 may be dissociated from Raf‐1(Figure 3b). Considering the significant accumulation of phospholipid peroxide observed in the midbrain of mice subjected to stress or CORT treatment, we conducted additional investigations to explore the alterations in the phospholipid oxidase ALOX15/PEBP1 complex. Indeed, CORT significantly enhanced the interaction between PEBP1 and ALOX15, whereas the Raf‐1/PEBP1 complex disappeared in a corresponding manner (Figure 3c). This effect was further confirmed through cellular immunofluorescence experiments (Figure 3d). We further investigated the effect of stress in a PD susceptibility model of stress‐induced A53T mice and found that, similar to CORT in PC12 cells, stress induced the dissociation of PEBP1 from Raf‐1 in the midbrain of mice, promoting the formation of more ALOX15/PEBP1 complexes (Figure 3e). Also, this finding was supported by the observation that CORT dose‐dependently increased PEBP1 and ERK phosphorylation, a phenomenon which was readily reversed by the PKC inhibitor staurosporine (STS) (Figure 3f,g). Further, STS prevented CORT‐induced lipid peroxidation in PC12 cells overexpressing A53T (Figure 3h,i). Together, these findings suggest that A53T‐induced upregulation of ALOX15 and CORT‐induced dissociation of PEBP1 may be accountable for subsequent lipid peroxidation.

### 3.4 ALOX15 is essential for PD susceptibility induced by stress

As an inhibitor of ferroptosis, ferrostatin‐1 (Fer‐1) is capable of inhibiting the phospholipid peroxidation by radical trapping and interfering with ALOX15/PEBP1 formation (Miotto et al., 2020). Our analysis of motor coordination and molecular biological detection found that Fer‐1 reversed the stress‐induced susceptibility to PD in A53T mice (Figure S4a–l). As expected, Fer‐1 attenuated stress‐induced lipid peroxidation and ferroptosis signaling pathway activation (Figure S4m,n), indicating that ferroptotic degeneration of dopaminergic neurons may plays a crucial role in the enhancement of PD susceptibility. Furthermore, we aimed to investigate the mechanism underlying the reduction of PD susceptibility by Fer‐1, focusing on its potential role in inhibiting the binding of ALOX15 and PEBP1. To this end, we performed immunoprecipitation experiments on midbrain tissue from A53T mice and found that Fer‐1 treatment reduced the level of stress‐induced ALOX15/PEBP1 complex (Figure S4o), suggesting that ALOX15/PEBP1 may be responsible for the stress‐induced PD susceptibility.

**FIGURE 4:**
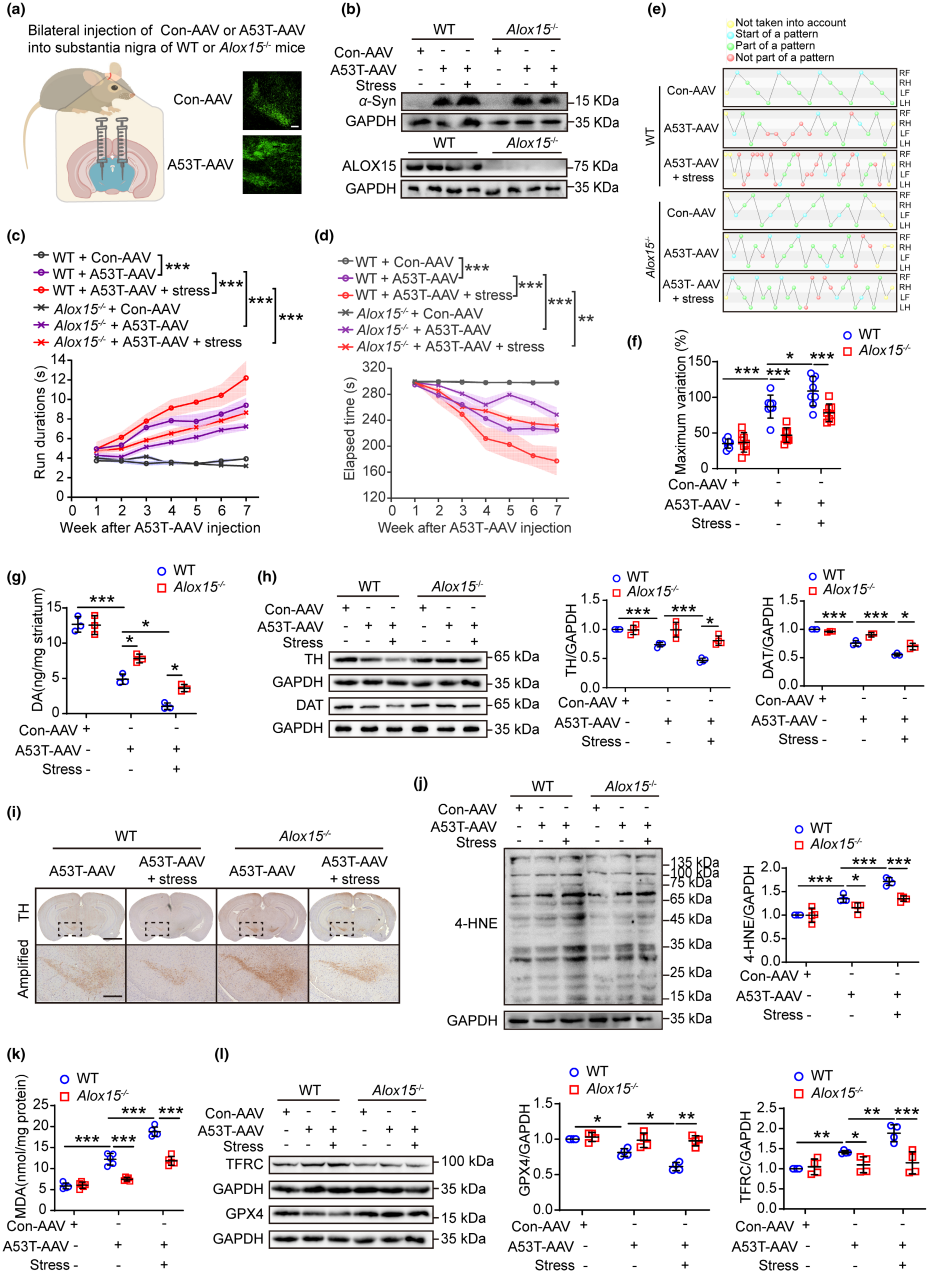
ALOX15 is required for parkinsonism and lipid peroxidation induced by stress. (a) Schematic diagram of showing that WT or 8‐week‐old *Alox15* ^−/−^ mice were bilaterally injected with Con‐AAV or A53T‐AAV into the substantia nigra (scale = 200 μm). (b) Western blotting of *α*‐Syn and ALOX15 in the midbrain of WT and *Alox15* ^−/−^ mice. (c–f) The pole test (c), rotarod test, (d) and CatWalk gait analysis (e, f) were used to evaluate the motor function after AAV‐loaded human *SNCA* injection in WT and *Alox15* ^*−/−*^ mice. RF, right front; RH, right hind; LF, left front; LH, left hind. Green balls: normal pattern. Red balls: abnormal pattern. Blue balls: the beginning of a walking cycle. Yellow balls: data that were excluded from the test (*n* = 8). (g) Dopamine was measured in the striatum (*n* = 3). (h) Western blotting (left) and quantification (right) of TH expression in the midbrain (*n* = 4) and DAT expression in striatum (*n* = 3). (i) IHC of coronal brain sections labeled with TH antibody and hematoxylin (upper, scale = 2 mm). The substantia nigra (dotted area) were amplified on bottom with scale = 500 μm (*n* = 3). (j) Western blotting of 4‐HNE‐protein adducts in the midbrain (*n* = 4). (k) The content of MDA in the midbrain was detected (*n* = 5). (l) Western blotting of ferroptosis‐related proteins in the midbrain (*n* = 4). All data represent mean ± SD. *p < 0.05, **p < 0.01, and ***p < 0.001, by two‐way ANOVA with Tukey (for c, d) and one‐way ANOVA with Tukey (for f, k), Bonferroni (for g, h, j) or LSD test (for l).

Since ALOX15 is essential for lipid peroxidation in membranes, *Alox15* knockout (KO) mice were employed for AAV‐mediated overexpression of A53T by stereotactic injection technique, in order to investigate the role of ALOX15 in stress‐induced PD susceptibility (Figure 4a,b). The absence of ALOX15 significantly attenuated impaired motor and coordination function in PD associated with A53T and stress, as demonstrated by pole test, rotarod test and gait analysis (Figure 4c–f; Figure S5a–j). Furthermore, compared with WT mice, *Alox15* KO mice showed significantly less damage to dopaminergic neurons (Figure 4g–i), as well as lower levels of lipid peroxidation in the midbrain in response to overexpressed A53T and stress (Figure 4j–l). Hence, these data support a conclusion that ALOX15/PEBP1 plays a crucial role in stress‐enhanced susceptibility in a PD mouse model.

### 3.5 Leonurine, an active alkaloid extracted from traditional Chinese medicine Herba leonuri, mitigated lipid peroxidation by interacting directly with ALOX15

**FIGURE 5:**
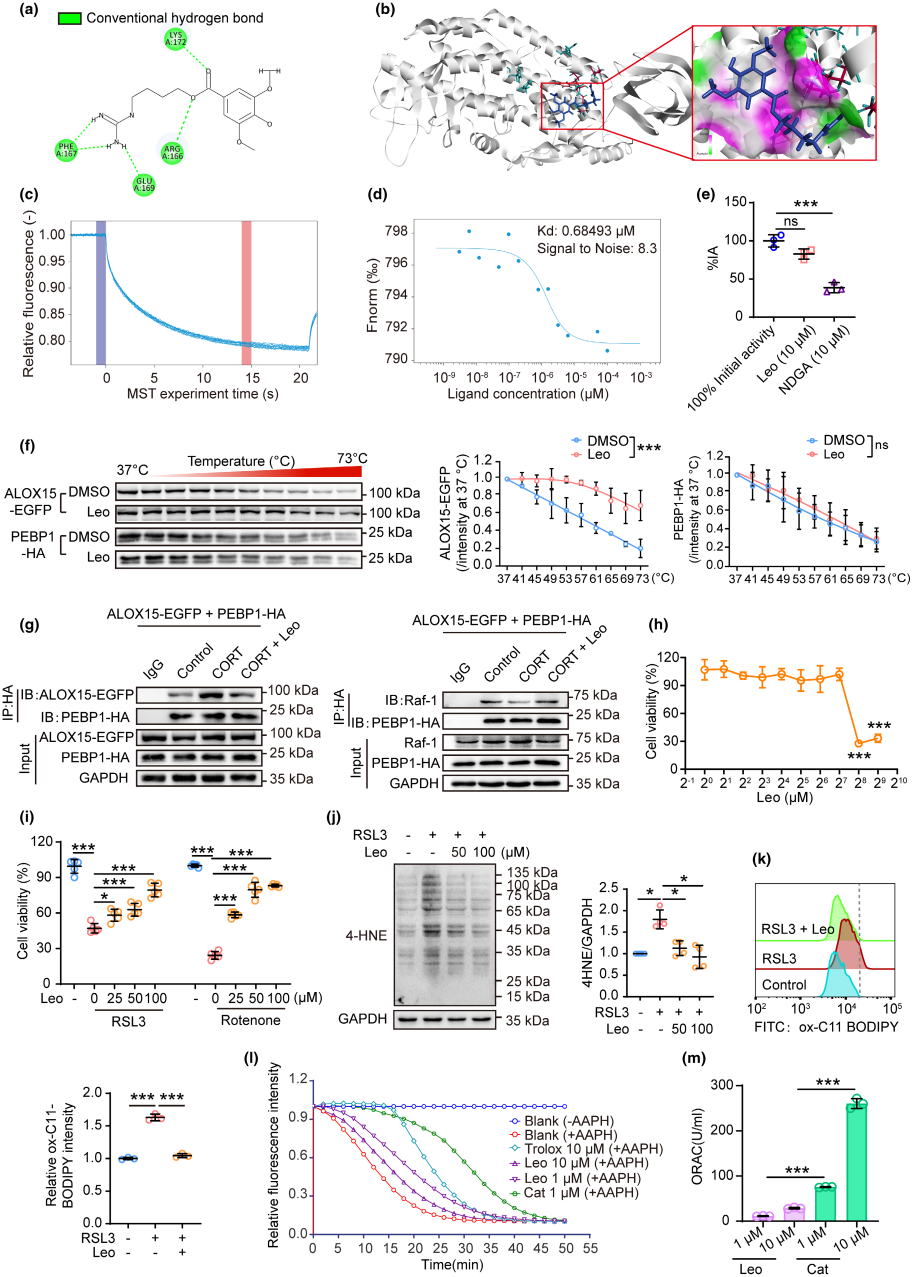
Leonurine suppresses the binding of ALOX15 to PEBP1 which inhibits ferroptosis. (a, b) Molecular docking of leonurine binding to ALOX15. (c, d) Leonurine–ALOX15 interaction was measured by microscale thermophoresis (MST). (e) Catalytic activity of ALOX15 was assessed by Lipoxygenase Inhibitor Screening Assay Kit (760700). Nordihydroguaiaretic acid (NDGA) served as a positive control drug (*n* = 3). (f) The interaction between ALOX15 and leonurine was determined by cellular thermal shift assay (CETSA). CETSA was performed in the lysates of ALOX15 and PEBP1‐overexpressing HEK293 cells (*n* = 3). (g) The interaction between PEBP1 and ALOX15 or Raf‐1 was examined by co‐IP in HEK‐293 cells overexpressing ALOX15‐EGFP and PEBP1‐HA (*n* = 3). CORT, 200 μM. Leonurine, 50 μM. (h) Cell viability of PC12 cells treated with leonurine alone at various concentrations (*n* = 5). (i) Cell viability of PC12 cells challenged with RSL3 (12.5 μM) or rotenone (1 μM) following administration of indicated concentration of leonurine for 24 h (*n* = 5). (j) The levels of 4‐HNE‐protein adducts were analyzed by western blot (*n* = 4). (k) Lipid peroxidation was detected by flow cytometry with C11 BODIPY (581/591) in PC12 cells (*n* = 3). RSL3, 12.5 μM. Leonurine, 50 μM. (l, m) Direct ROS scavenging capacity of leonurine was evaluated by oxygen radical absorbance capacity (ORAC) assay (*n* = 3). All data represent mean ± SD. *p < 0.05, **p < 0.01, and ***p < 0.001, by two‐way ANOVA with Tukey (for f) and one‐way ANOVA with Dunnett T3 test (for e, h, j) and Tukey (for i).

As reported in one of our previous studies, TCM preparation named Tianma Gouteng granule (TG) alleviated behavioral disturbances in PD models by inhibiting ALOX15‐mediated lipid peroxidation (Jiang et al., 2020). To discover potential compounds, we docked TG's main components with ALOX15 (Table S2). Leonurine, out of the 17 compounds studied, exhibited the greatest potential for interaction with ALOX15 through the formation of hydrogen bonds with Arg166, Glu169, Lys172, and Phe167, which are located close to the site where PEBP1 binds (Anthonymuthu et al., 2021) (Figure 5a,b and TableS2). Furthermore, we applied the MST assay and cell thermal shift assay (CETSA) to provide additional evidence that leonurine directly bound ALOX15 rather than PEBP1 (Figure 5c,d,f). Intriguingly, leonurine did not inhibit ALOX15 enzymatic activity (Figure 5e), but instead significantly disrupted ALOX15/PEBP1 interaction (Figure 5g). As a consequence, leonurine dose‐dependently rescued ferroptosis and lipid peroxidation induced by RSL3 (Figure 5h–k) or by rotenone, even when ALOX15 is overexpressed (Figure S6a–d). Next, we sought to determine the effects of leonurine on the CORT treatment in *SNCA*‐overexpressed PC12 cells. As a result of CORT‐induced increases in lipid peroxidation products 4‐HNE and MDA, as well as activation of TFRC and *Ptgs2*, and decreases in TH, leonurine significantly reversed these changes (Figure S6e–h), suggesting that leonurine protected against the peroxidative damage induced in neurons by stress. Notably, the protective effects of leonurine were found to be independent of GPX4 (Figure S6g,i). To investigate the mechanism underlying the neuroprotective effects of leonurine, we performed the ORAC assay to determine whether leonurine can directly reduce reactive oxygen species (ROS). Our results showed that leonurine chelated significantly less ROS than the free radical scavengers Trolox or catechin (Figure 5l,m), suggesting that leonurine may act as a neuroprotective agent by preventing ALOX15 from oxidizing membrane lipids and inducing lipid peroxidation.

### 3.6 Leonurine relieves the susceptibility of mice under stress to PD

**FIGURE 6:**
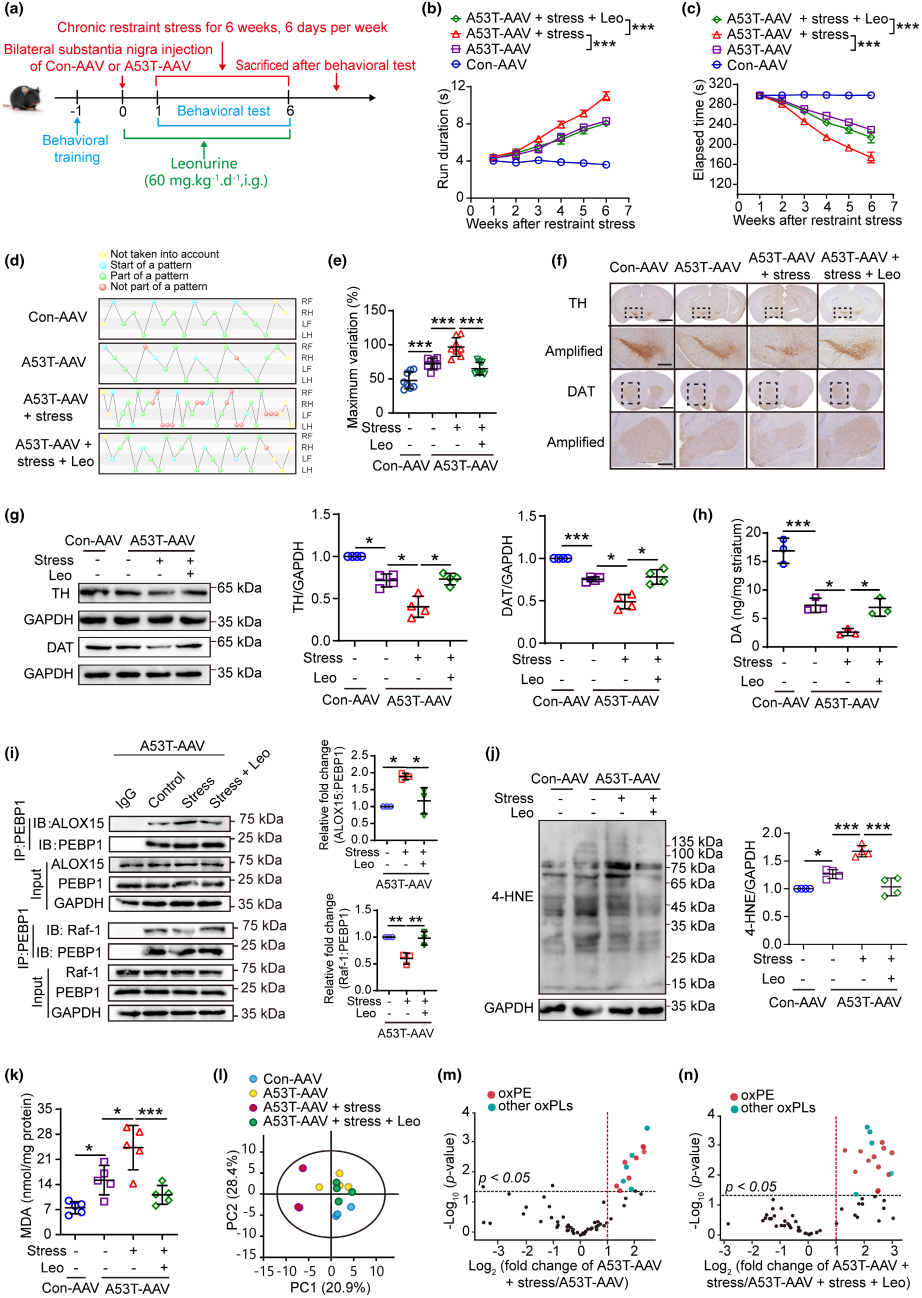
Leonurine attenuates stress‐induced parkinsonism by inhibiting ferroptotic neuronal death. (a) Schematic diagram of the experimental design. The mice were bilaterally injected with control AAV or A53T‐AAV into the substantia nigra and given restraint stress, leonurine treatment for 6 weeks postoperatively, during which behavioral tests were performed. (b–e) The pole test (b), rotarod test (c), and CatWalk gait analysis (d, e) were used to evaluate the motor function. RF, right front; RH, right hind; LF, left front; LH, left hind. Green balls: normal pattern. Red balls: abnormal pattern. Blue balls: the beginning of a walking cycle. Yellow balls: data that were excluded from the test (*n* = 8). (f) IHC of coronal brain sections labeled with TH antibody or DAT antibody and hematoxylin (scale = 2 mm). The substantia nigra and striatum (dotted area) were amplified on bottom with scale = 500 μm. The amplified images of DAT were rotated 90° for display. (n = 3). (g) Western blotting and quantitative analysis of TH expression in midbrain and DAT expression in striatum (*n* = 4). (h) The dopamine was measured in striatum (*n* = 3). (i) The binding of PEBP1 to ALOX15 or Raf‐1 after treatment with stress or leonurine was detected by co‐IP in A53T‐AAV mice (*n* = 3). (j) Western blotting (left) and quantitative analysis (right) of 4‐HNE‐protein adducts (*n* = 4). (k) The content of MDA was detected in midbrain (*n* = 5). (l–n) Data of oxidized phospholipids were interpreted by principal component analysis (PCA) and volcano plots. All data represent mean ± SD. *p < 0.05, **p < 0.01, and ***p < 0.001, by two‐way ANOVA with Tukey (for b, c) and one‐way ANOVA with Tukey (for e, k), Dunnett T3 (for g), Bonferroni (for h–j).

To evaluate the efficacy of leonurine, a PD mouse model of MPTP was used to receive leonurine treatment according to the recommended doses (Jia et al., 2017), with rasagiline serving as a positive control. No effect was observed on the weight of mice as a result of the treatments (Figure S7a). Based on the results of the pole test, rotarod test, and gait analysis, leonurine significantly protected mice from MPTP‐induced parkinsonism at both doses (Figure S7b–k). Leonurine at a higher dose was more effective in reversing the MPTP‐induced loss of dopaminergic neurons (Figure S7l–n). Therefore, the higher dose of leonurine was applied in the subsequent experiments using the previously described PD‐susceptible mouse model, which was bilaterally injected with A53T‐carrying AAV into the substantia nigra and then underwent stress (Figure 6a). Compared to the untreated PD‐susceptible mice, leonurine significantly improved the motor coordination as demonstrated by the behavioral analysis (Figure 6b–e; Figure S8a–h). Aside from this, leonurine was shown to exhibit significant protective effects against dopaminergic neuron loss (Figure 6f–h) and lipid peroxidation associated with PD (Figure 6j,k), which was also verified in an MPTP‐induced mouse model (Figure S7o,p). We further investigated the effect of leonurine on the binding of ALOX15 and PEBP1 in mice. Our results showed that leonurine significantly reduced the binding of ALOX15 and PEBP1, while tending to increase the binding of PEBP1 to Raf‐1 (Figure 6i). Analyses using LC–MS/MS of oxidized phospholipids revealed that leonurine significantly reduced the accumulation of membrane lipid peroxides caused by A53T and stress (Figure 6l–n; Figure S8l). Moreover, leonurine treatment decreased the level of TFRC and *Ptgs2*, but did not affect the expression of GPX4, appeared not to be involved in the action of leonurine (Figure S8i–k). Collectively, our findings suggest that leonurine may reduce the susceptibility to PD in mice by inhibiting lipid peroxidation.

## 4 DISCUSSION

Increasing aging and environmental pollution are contributing to the global burden of PD, which affects 2% of people over the age of 60 (Tysnes & Storstein, 2017). With the dramatic increase in life pressure, long‐term psychological stress, which may refer to “emotional stress,” could cause people to go into a disease‐susceptible state similar to sub‐health. It may be characterized by a decrease in physical strength, function, and adaptability; however, the exact diagnostic criteria are difficult to define. It results from a breakdown in the dynamic balance system that combines biological and psychological factors, including individual life events and psychosomatic symptoms (Pan et al., 2020). In fact, modern medical research has also found evidence of psychological stress increasing susceptibility to PD, and clinical studies have showed that stressful life events may contribute to the development of PD (Ibrahimagic et al., 2016; Sieurin et al., 2018). In our study, we employed a chronic restraint stress model to simulate a state of psychological stress, and our finding demonstrated that this type of stress does promote susceptibility to PD, which may explain the different “susceptibility” to PD among individuals with the same PD mutant genotype in different stress states.

Previous studies have demonstrated potential mechanisms for the connector between stress and neurodegeneration related to the immune system and oxidative stress. Pro‐inflammatory mediators released by brain immune cells in response to stress diminish neuronal structure and function, and increase inflammatory gene expression and lipid peroxidation (Peña‐Bautista et al., 2020). Further, stress induces microglia activation and oxidative stress, which triggers dopaminergic and noradrenergic neurodegeneration and exacerbates PD progression (Sugama et al., 2016). While researchers are able to associate stress with altered corticosteroid release from the HPA axis (Herman et al., 2016), the exact mechanism responsible for this remains unclear. In this study, we precisely described the molecular mechanism contribute to stress and corticosteroids‐associated dopaminergic neuron loss in the substantia nigra pars compacta and striatum in synaptic terminal which causes PD‐like behavioral disturbances in A53T mouse models. Large amounts of lipid peroxides (4‐HNE and MDA) were observed in the midbrain of A53T mice that had been subjected to stress and CORT. The mechanism underlying PD susceptibility was further explored using LC–MS/MS‐based phospholipidomics, which revealed an involvement of the lipid peroxidation occurred in the plasma membrane, especially in oxPEs. OxPEs have been identified serving as a trigger of ferroptosis, a newly established unconventional form of cell death (Anthonymuthu et al., 2018; Do Van et al., 2016; Kagan et al., 2017; Wenzel et al., 2017). There appears to be a link between ferroptosis and neuronal loss in PD. The hallmark features of ferroptosis include iron overload, membrane lipid peroxidation, reduced levels of GSH and Coenzyme Q10, and decreased expressions of xCT (SLC7A11) and DJ‐1, all of which are pathologically characterized in PD (Mahoney‐Sánchez et al., 2021). It has been demonstrated that stress increased lipid peroxidation products in rat brains (Herbet et al., 2017). Besides, the corticosteroids‐derived oxidative stress were reported to accelerate the alteration of genes or protein pathways that contribute to ferroptosis (Yang et al., 2021). Interestingly, ferroptotic markers and signaling pathways were identified in the midbrain of A53T mice that had either been subjected to stress or CORT to determine how ferroptosis contributed to the loss of dopaminergic neurons in vivo. It was further demonstrated in PC12 cells that CORT dose‐dependently increased the sensitivity to ferroptosis inducer RSL3, and this was even more pronounced when A53T was overexpressed. Furthermore, we demonstrated that scavenging membrane lipid peroxides by Fer‐1 was effective in treating parkinsonism that is exacerbated by stress. Specifically, we demonstrated that Fer‐1 attenuated lipid peroxidation in stress‐induced PD susceptibility by inhibiting PEBP1 and ALOX15, which was recruited to the plasma membrane by PEBP1 to catalyze the oxidation of polyunsaturated fatty acids in phospholipids into their corresponding hydroperoxyl derivatives. While Fer‐1 is known to have potent free radical scavenging activity, it remains unclear whether the protective effect against PD is due to this activity or its ability to inhibit the ALOX15/PEBP1 complex, or both.

In fact, there are numerous factors that can contribute to oxPEs changes, including the synthesis of PE, the activity of oxidative enzymes such as ALOX15 and CYP450, and metabolism and scavenging of oxPEs, which involves many enzymes and proteins such as PLA2G6, GPX4, and endogenous free radical scavengers (Stockwell, 2022). Therefore, the changes in oxPEs induced by stress and CORT may be different, given that restraint stress involves multiple changes in the central nervous system, hormonal fluctuations, and possible hypoxia, which cannot be fully replicated by CORT alone. In our study, we have revealed one of the possible mechanisms. Our results demonstrated a significant increase in oxPEs in the midbrain of mice subjected to stress or treated with CORT. Further analysis revealed that both stress and CORT treatment promoted the binding of the ALOX15/PEBP1 complex, which may contribute to the observed elevation in oxPEs.

The lipoxygenases, including ALOX15, are a family of enzymes which initiate lipid peroxidation of membranes as well as synthesis of signaling molecules by di‐oxygenating unsaturated fatty acids, such as arachidonic acid (AA) and linoleic acid (LA) (Maccarrone et al., 2001). We found that in the midbrain of A53T mice, ALOX15 expression was increased and PEBP1 was found to form a complex with ALOX15, encouraging lipid peroxidation and facilitating remarkable ferroptosis loss of dopaminergic neurons. In accordance with the results obtained, CORT indeed promoted the dissociation of PEBP1 from Raf‐1 through activation of the PKC pathway, after which PEBP1 began searching for a new partner. We next focused on corroborating the key role of ALOX15 in PD pathogenesis. Specially, as the onset of A53T transgenic mice is long (9–16 months), scattered, and has a low probability, and SNCA does not require systemic expression in order to cause PD pathogenesis, we injected A53T‐AAV through bilateral midbrain stereotaxic localization to induce the PD‐like model. In our research, A53T‐AAV mice lacking ALOX15 demonstrated decreased susceptibility to stress‐induced neurotoxicity as a result of inhibitory effects on lipid peroxidation and neuronal ferroptosis, suggesting an inflammation‐linked mechanism for PD susceptibility.

Compounds derived from herbs have demonstrated good neuroprotective efficacy both in vivo and in vitro, and screening through its compound library may provide a promising method for developing a new drug. Herbal medicine has gradually gained attention as a means of regulating lipid metabolism and reducing lipid oxidation in recent decades. In the *Chinese Pharmacopoeia*, *Leonuri* is commonly used as a gynecological drug to regulate menstruation and promote blood circulation. Among the approximately 200 chemical compounds in *Leonurus japonicus*, alkaloids are the most important biologically active components. Particularly, leonurine is one of the most crucial constituents which has been shown to have a wide range of biological activities, including anti‐inflammatory (Song et al., 2015) and neuroprotective properties (Liu et al., 2012). While leonurine has been discovered to alleviate depression‐like behaviors in a stress model via suppressing the activation of NF‐*κ*B pathway (Jia et al., 2017), its detailed mechanism of action has not been fully explored. In this study, leonurine was identified from TG and was able to protect dopaminergic neurons from stress and MPTP by directly interacting with ALOX15 and preventing the formation of ALOX15/PEBP1 complexes, providing insights into the therapeutic potential of ALOX15/PEBP1 for the targets of PD treatment.

**FIGURE 7:**
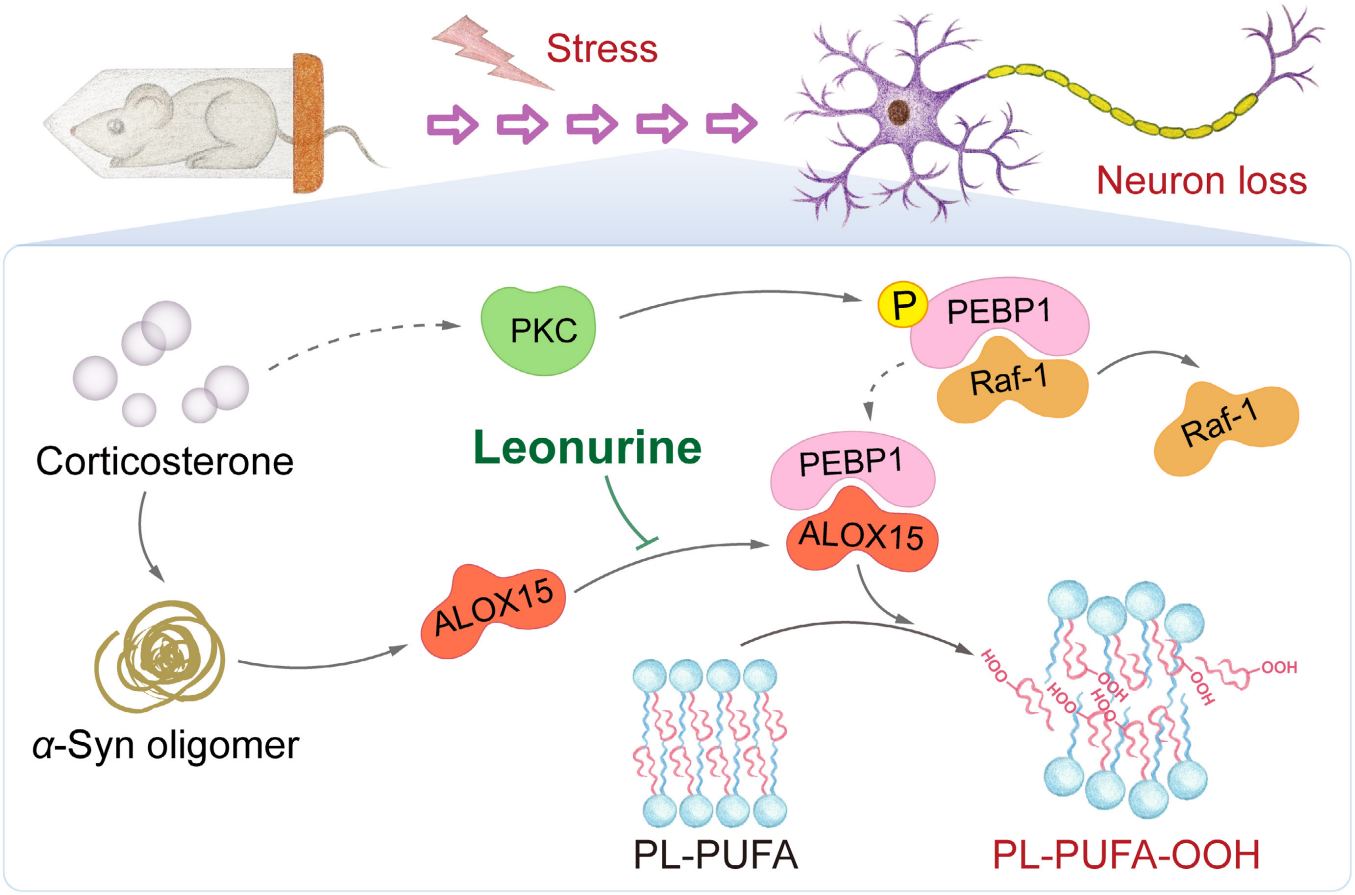
Stress drives the loss of dopaminergic neurons via membrane lipid peroxidation. Mechanistically, stress drives the HPA axis to release corticosteroids, especially corticosterone, and corticosterone activates the PKC pathway, which dissociates PEBP1 from Raf‐1 to bind ALOX15, which assembles into a complex that catalyzes the phospholipid peroxidation and promotes the ferroptosis sensitivity of dopaminergic neurons. Synergistically, blocking the assembly of the ALOX15/PEBP1 complex through leonurine attenuates phospholipid peroxidation, thereby reducing chronic stress‐induced susceptibility to Parkinson's disease.

Collectively, the mechanism of stress that contributes to the susceptibility to PD is identified by triggering the release of the stress hormone CORT, which facilitates the interaction between ALOX15 and PEBP1 and causes lipid peroxidation in the plasma membrane, resulting in susceptibility to ferroptosis of the dopamine neurons. A significant reduction in PD susceptibility was achieved by inhibiting the formation of the complex between ALOX15 and PEBP1 with leonurine, a natural alkaloid derived from the traditional Chinese medicine herb *leonuri* (Figure 7).

## AUTHOR CONTRIBUTIONS

Xiao‐Min Lin conducted most of the in vitro and in vivo experiments, analyzed the data, prepared the figures, and wrote the manuscript. Ming‐Hai Pan and Jie Sun conducted most of the in vivo experiments, analyzed the data, and prepared the figures. Meng Wang, Zi‐Han Huang, and Rui‐Ting Huang assisted in conducting in vivo experiments and acquired data. Guan Wang assisted in conducting the molecular docking experiment. Rong Wang, Hai‐Biao Gong, and Wan‐Yang Sun conducted the LC–MS/MS‐based experiments and acquired data. Feng Huang, Hai‐Zhi Liu, Hiroshi Kurihara, and Yi‐Fang Li supervised and advised the project. Wen‐Jun Duan supervised and advised the project, analyzed and approved the data, and revised the manuscript. Rong‐Rong He designed and supervised the project, and approved the manuscript. All the authors have read and approved the paper.

## ACKNOWLEDGMENTS

This work was supported, in part, by the National Natural Science Foundation of China (82125038, 82274123, 82274403), Guangdong Basic and Applied Basic Research Foundation (2021B1515120023, 2023B1515020066, 2023B1515020020, 2023B1515040016), Guangzhou Basic and Applied Basic Research Foundation (202102020001), the Local Innovative and Research Teams Project of Guangdong Pearl River Talents Program (2017BT01Y036) and GDUPS (2019), the Science and Technology Program of Guangzhou (907158833068), the Innovation Team Project of Guangdong Provincial Department of Education (2020KCXTD003).

## CONFLICT OF INTEREST STATEMENT

The authors declare that they have no conflict of interest.

## Supporting information


**Data S1.** Supporting Information.

## Data Availability

The data that support the findings of this study are available from the corresponding author upon reasonable request.
