# Supplementary material for: Membrane phospholipid peroxidation promotes loss of dopaminergic neurons in psychological stress‐induced Parkinson's disease susceptibility
Source: Aging Cell. 2023 Aug 25;22(10):e13970. doi: 10.1111/acel.13970 (PMC10577563; doi:10.1111/acel.13970)
Supplement: Supplementary file 1 — Data S1. Supporting Information. [file ACEL-22-e13970-s001.docx]

Supporting Information

**Membrane phospholipid peroxidation promotes loss of dopaminergic neurons in psychological stress-induced Parkinson's disease susceptibility**

Xiao-Min Lin^1^, Ming-Hai Pan^1^, Jie Sun^1^, Meng Wang^1^, Zi-Han Huang^1^, Guan Wang^4^, Rong Wang^3^, Hai-Biao Gong^1^, Rui-Ting Huang^2^, Feng Huang^3^, Wan-Yang Sun^1^, Hai-Zhi Liu^*,1^, Hiroshi Kurihara^1^, Yi-Fang Li^1^, Wen-Jun Duan^*,1^, and Rong-Rong He^*,1,2,3^

**The file include:**

Figure S1. Stress accelerates parkinsonism and lipid peroxidation in A53T mice.

Figure S2. Aggravation of PD motor impairment by CORT in A53T mice.

Figure S3. CORT promotes lipid peroxidation and *α*-synuclein accumulation *in vitro*.

Figure S4. Lipid peroxidation is responsible for stress-induced parkinsonism and dopaminergic damages.

Figure S5. The deficiency of ALOX15 reversed the motor impairment induced by stress.

Figure S6. Leonurine protects cells via inhibiting lipid peroxidation.

Figure S7. Leonurine alleviates behavioral disorder in MPTP-induced PD mouse model.

Figure S8. Leonurine mitigates stress-induced behavioral disorder in A53T-AAV PD mouse model.

Table S1. Primers sequences for RT-PCR.

Table S2. The docking parameters of TG compounds.

Table S3. List of AAVs and plasmids.


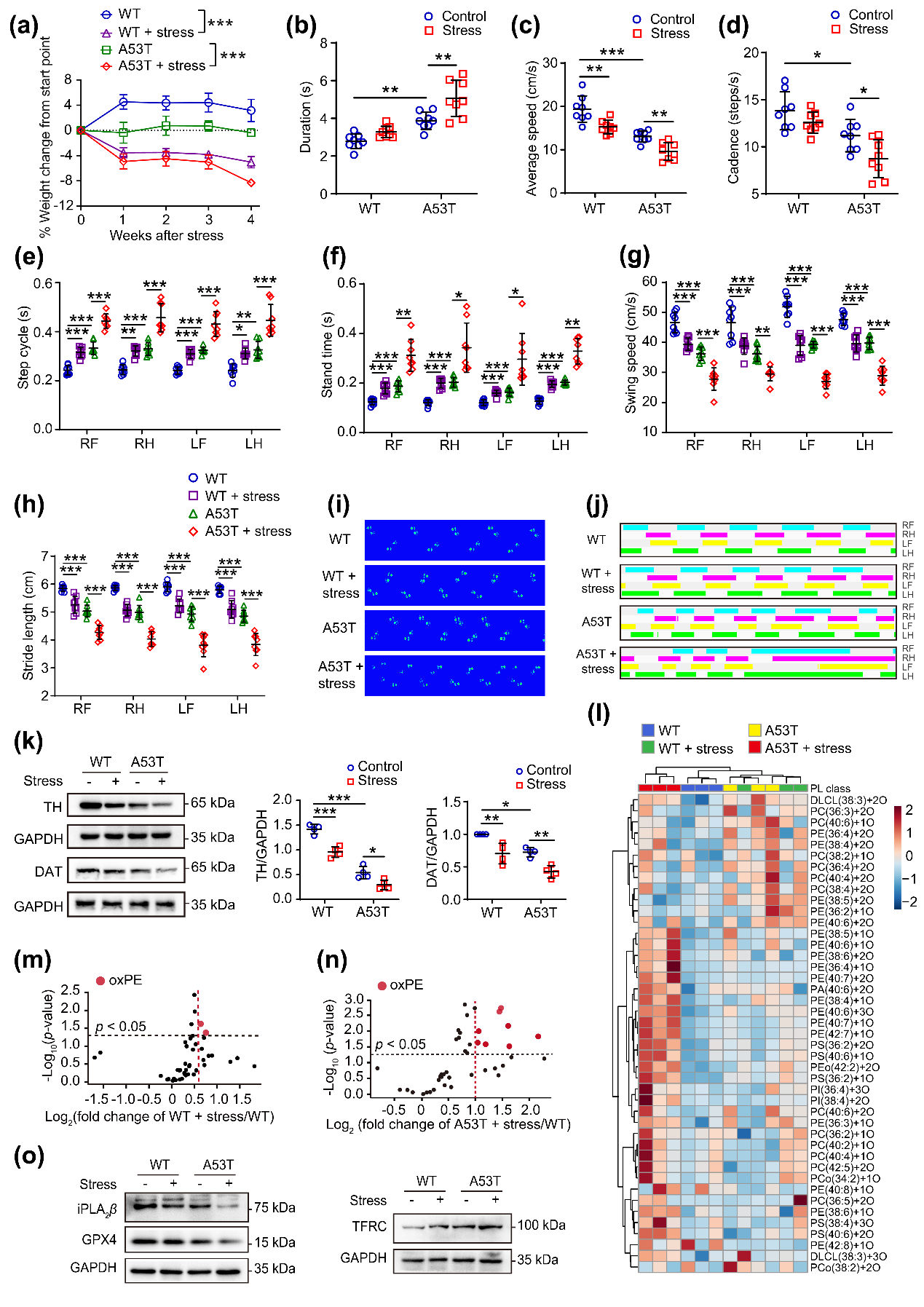


**Figure S1.** Stress accelerates parkinsonism and lipid peroxidation in A53T mice. (a) Percentage change from start point in body weight of mice (*n* = 8). (b-h) Disordered motor coordination of mice was assessed by CatWalk gait analysis including the duration of a run (b), average speed (c), cadence (d), step cycle (e), stand time (f), swing speed (g) and stride length (h) (*n* = 8). (i, j) The gait pattern, referring to the order in which the mice complete a sequence of walking, was visualized as footprint view (i) and footprint length (j) (*n* = 8). RF, right front. RH, right hind. LF, left front. LH, left hind. (k) The TH expression in midbrain and DAT expression in striatum were determined by western blot (*n* = 4). (l) Data of the oxidized phospholipids in the midbrain were extracted and displayed as heatmap. (m, n) The changes in the levels of oxygenated phospholipids were displayed by volcano plots. (o) Western blotting of ferroptosis-related proteins in the midbrain (*n* = 4). All data represent mean ± SD. ^*^*p* < 0.05, ^**^*p* < 0.01 and ^***^*p* < 0.001, by two-way ANOVA with Tukey (for a) and one-way ANOVA with Tukey (for b-h) or Bonferroni test (for k).


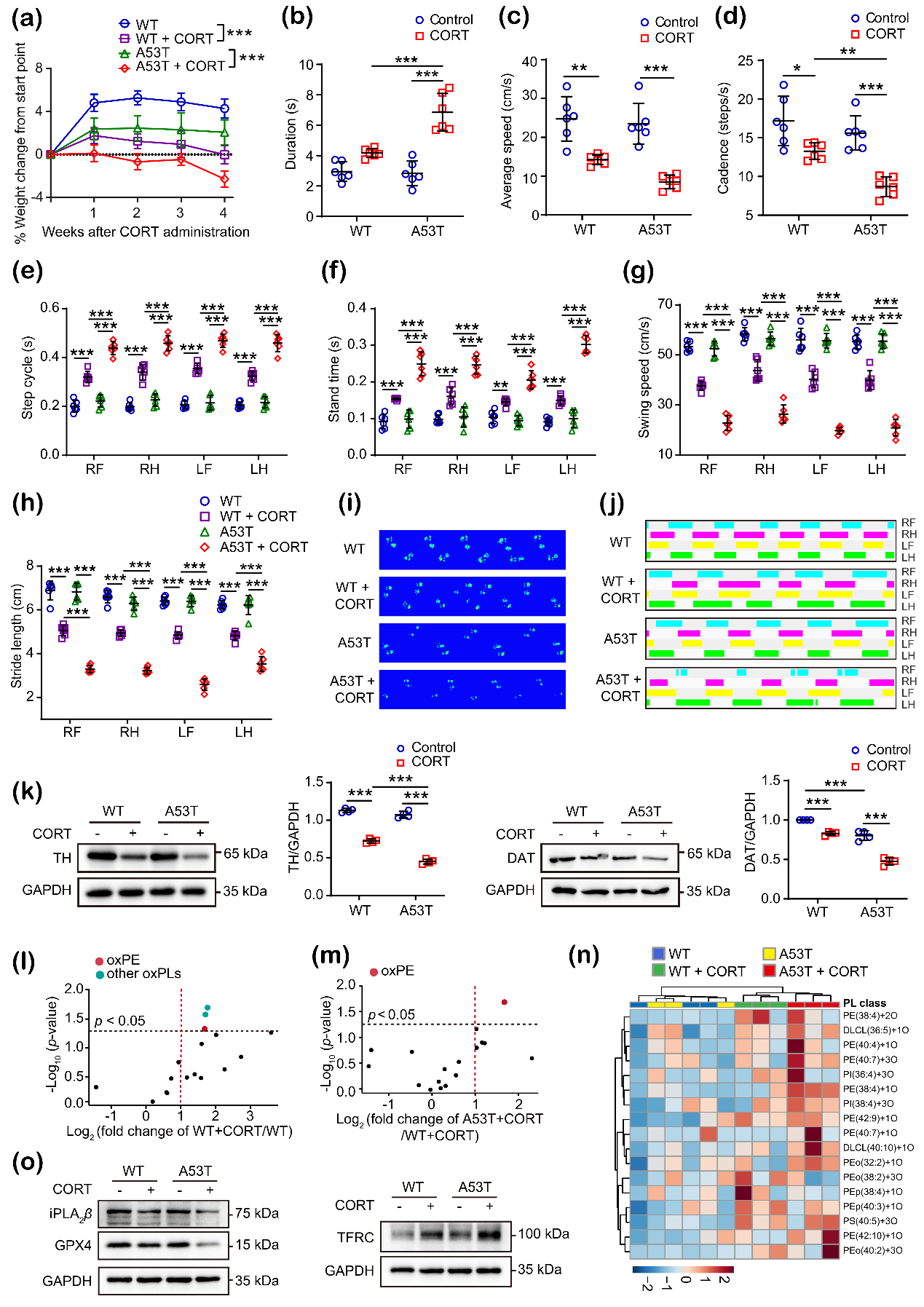


**Figure S2.** Aggravation of PD motor impairment by CORT in A53T mice. (a) Percentage change from start point in body weight of mice (*n* = 6). (b-h) Disordered motor coordination of mice was assessed by gait analysis including the duration of a run (b), average speed (c), cadence (d), step cycle (e), stand time (f), swing speed (g) and stride length (h). (i, j) The gait pattern, referring to the order in which the mice complete a sequence of walking, was visualized as footprint view (i) and footprint length (j) (*n* = 6). RF, right front. RH, right hind. LF, left front. LH, left hind. (k) The TH expression in midbrain and DAT expression in striatum were determined by western blot (*n* = 4). (l, m) The changes in the levels of oxygenated phospholipids were displayed by volcano plots. (n) Data of the oxidized phospholipids in the midbrain were extracted and displayed as heatmap. (o) Western blotting of ferroptosis-related proteins in midbrain (*n* = 4). All data represent mean ± SD. ^*^*p* < 0.05, ^**^*p* < 0.01 and ^***^*p* < 0.001, by two-way ANOVA with Tukey (for a) and one-way ANOVA with Tukey (for b-h) or Bonferroni test (for k).


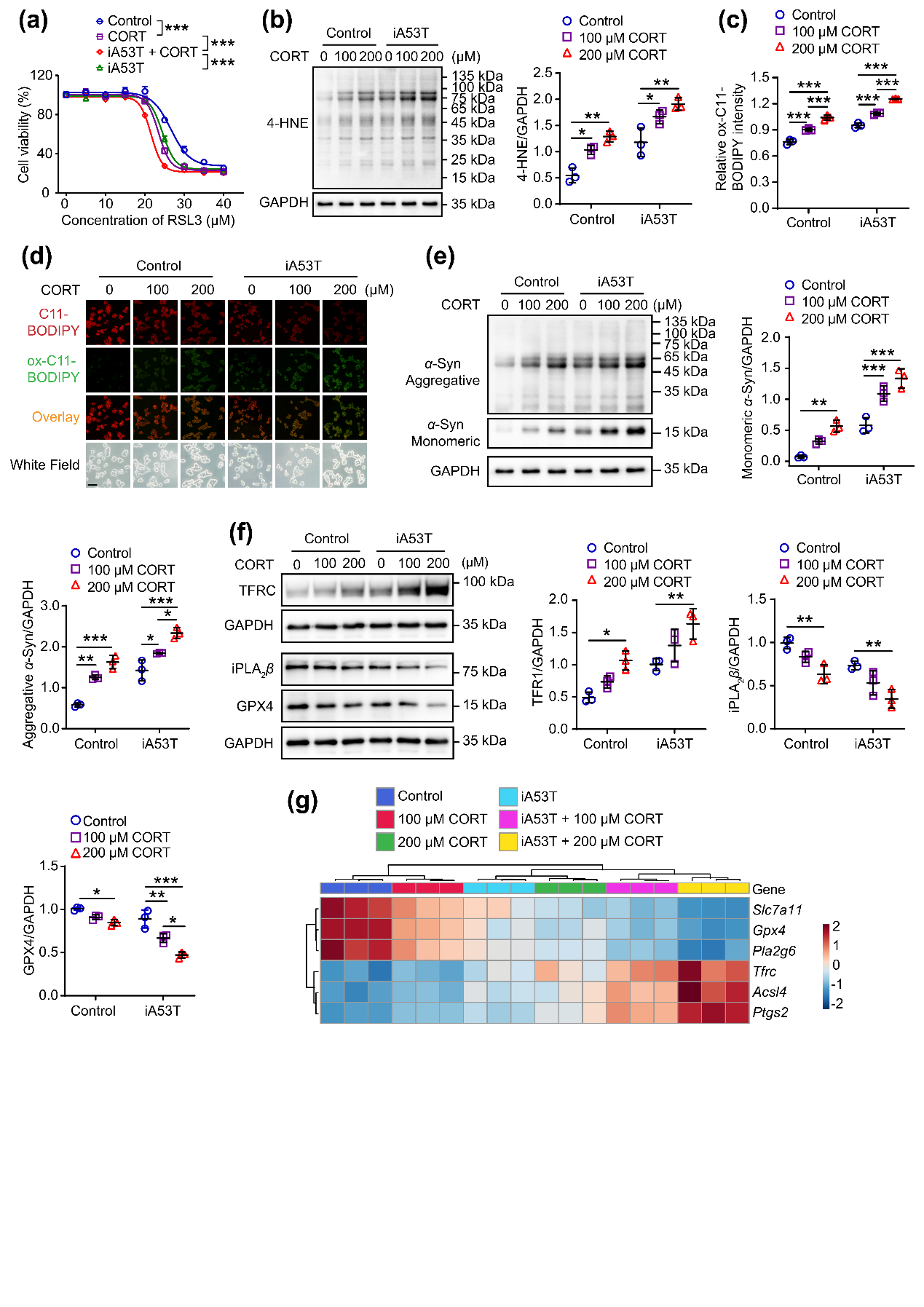


**Figure S3.** CORT promotes lipid peroxidation and *α*-synuclein accumulation *in vitro*. (a) Cell viability of PC12 cells under indicated concentrations of RSL3 following treatment with 200 μM CORT for 24 h (*n* = 3). (b) Western blotting (left) and quantitative analysis (right) of 4-HNE-protein adducts (*n* = 3). (c, d) Lipid peroxidation was detected by flow cytometry with C11 BODIPY (581/591) (c) and fluorescence microscope imaging (d). Scale = 50 μm. (e, f) Western blotting and quantitative analysis of α-synuclein and ferroptosis pathway-related proteins (*n* = 3). (g) Heatmap of ferroptosis pathway-related genes affected by CORT. All data represent mean ± SD. ^*^*p* < 0.05, ^**^*p* < 0.01 and ^***^*p* < 0.001, by two-way ANOVA with Bonferroni (for a) and one-way ANOVA with Bonferroni test (for b, c, e, f).


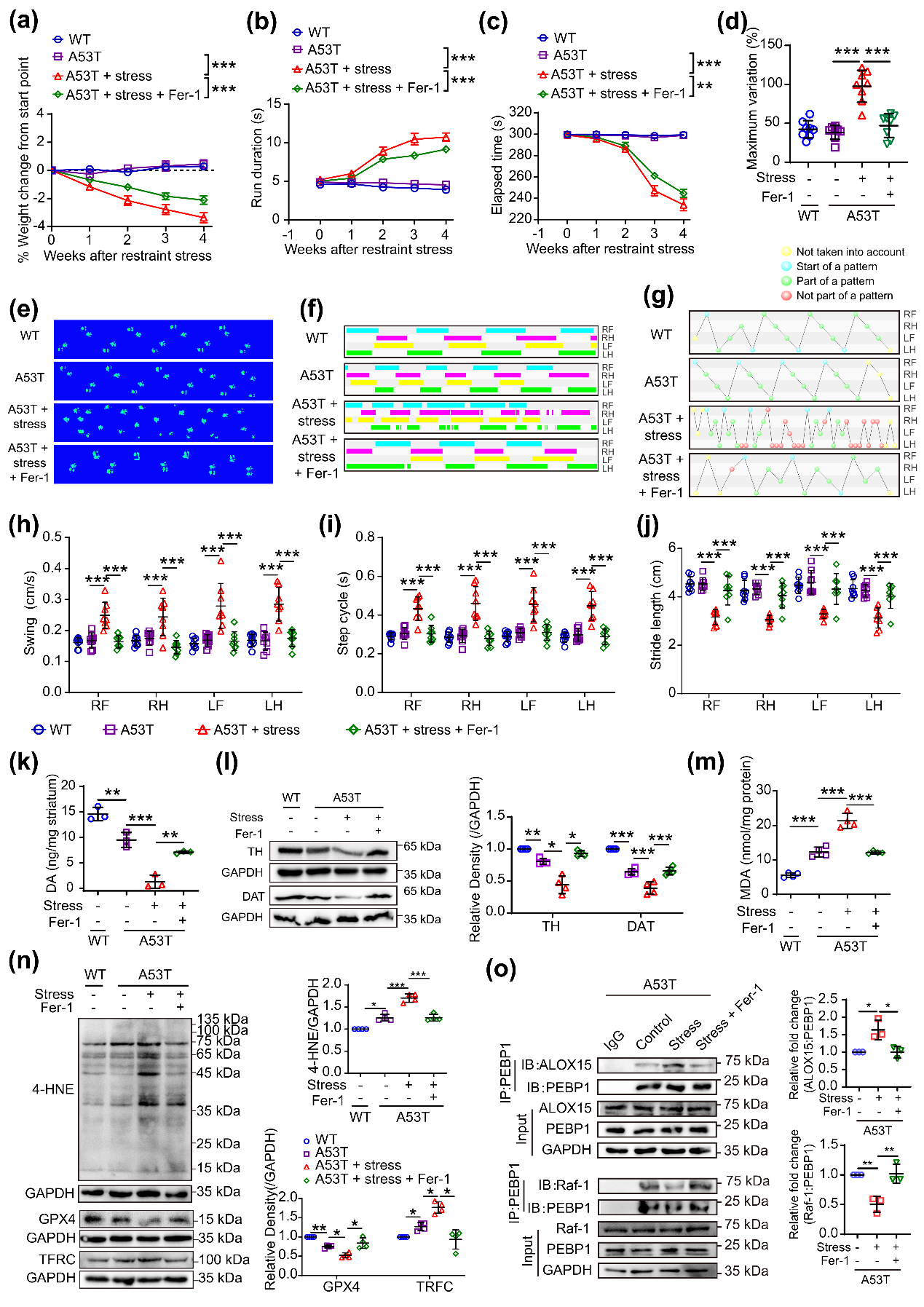


**Figure S4.** Lipid peroxidation is responsible for stress-induced parkinsonism and dopaminergic damages. (a) Percentage change from start point in body weight of mice (*n* = 8). (b-j) The pole test (b), rotarod test (c) and gait analysis (d-j) were used to evaluate the motor function of WT and A53T mice after restraint stress and ferrostatin-1 treatment (0.1 mg/kg, *i.c.v.*). RF, right front. RH, right hind. LF, left front. LH, left hind. Green balls: normal pattern. Red balls: abnormal pattern. Blue balls: the beginning of a walking cycle. Yellow balls: data that were excluded from the test (*n* = 8). (k) The dopamine was measured in striatum (*n* = 3). (l) Western blotting (left) and quantitative analysis (right) of TH expression in midbrain and DAT expression in striatum (*n* = 4). (m) The content of MDA was detected in midbrain (*n* = 4). (n) The levels of 4-HNE-protein adducts and ferroptosis pathway-related proteins were analyzed by western blot (*n* = 4). (o) The binding of PEBP1 to ALOX15 or Raf-1 after treatment with stress or Fer-1 was detected by co-IP in A53T-AAV mice (*n* = 3). All data represent mean ± SD. ^*^*p* < 0.05, ^**^*p* < 0.01 and ^***^*p* < 0.001, by two-way ANOVA with Tukey (for a-c) and one-way ANOVA with Tukey (for d, h-j) or Bonferroni test (for k-o).


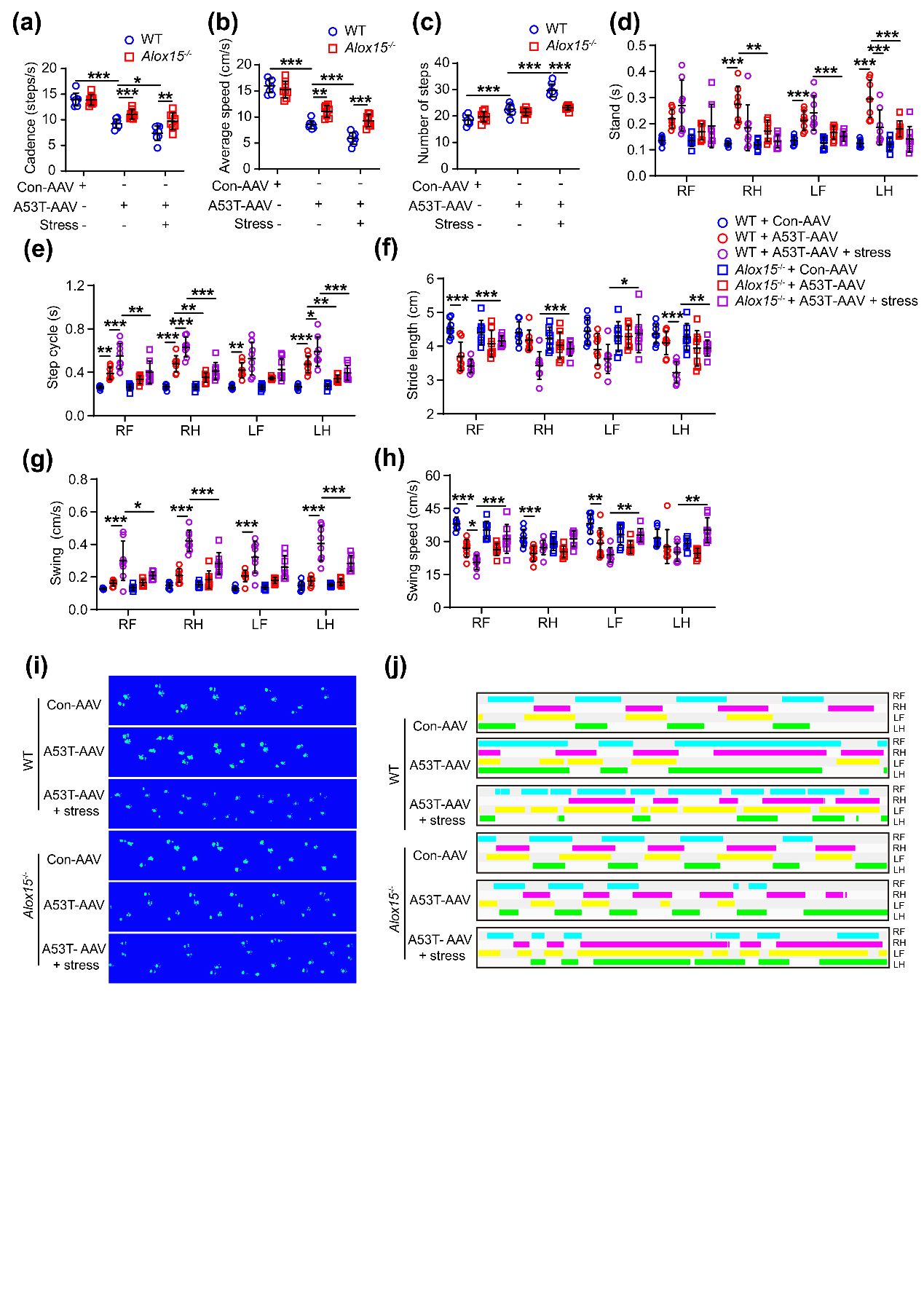


**Figure S5.** The deficiency of ALOX15 reversed the motor impairment induced by stress. (a-h) Disordered motor coordination of mice was assessed by CatWalk gait analysis including cadence (a), average speed (b), number of steps (c), stand (d), step cycle (e), stride length (f), swing (g) and swing speed (h) (*n* = 8). (i, j) The gait pattern, referring to the order in which the mice complete a sequence of walking, was visualized as footprint view and footprint length. RF, right front. RH, right hind. LF, left front. LH, left hind. (*n* = 8). All data represent mean ± SD. ^*^*p* < 0.05, ^**^*p* < 0.01 and ^***^*p* < 0.001, by one-way ANOVA with Tukey (a-h).


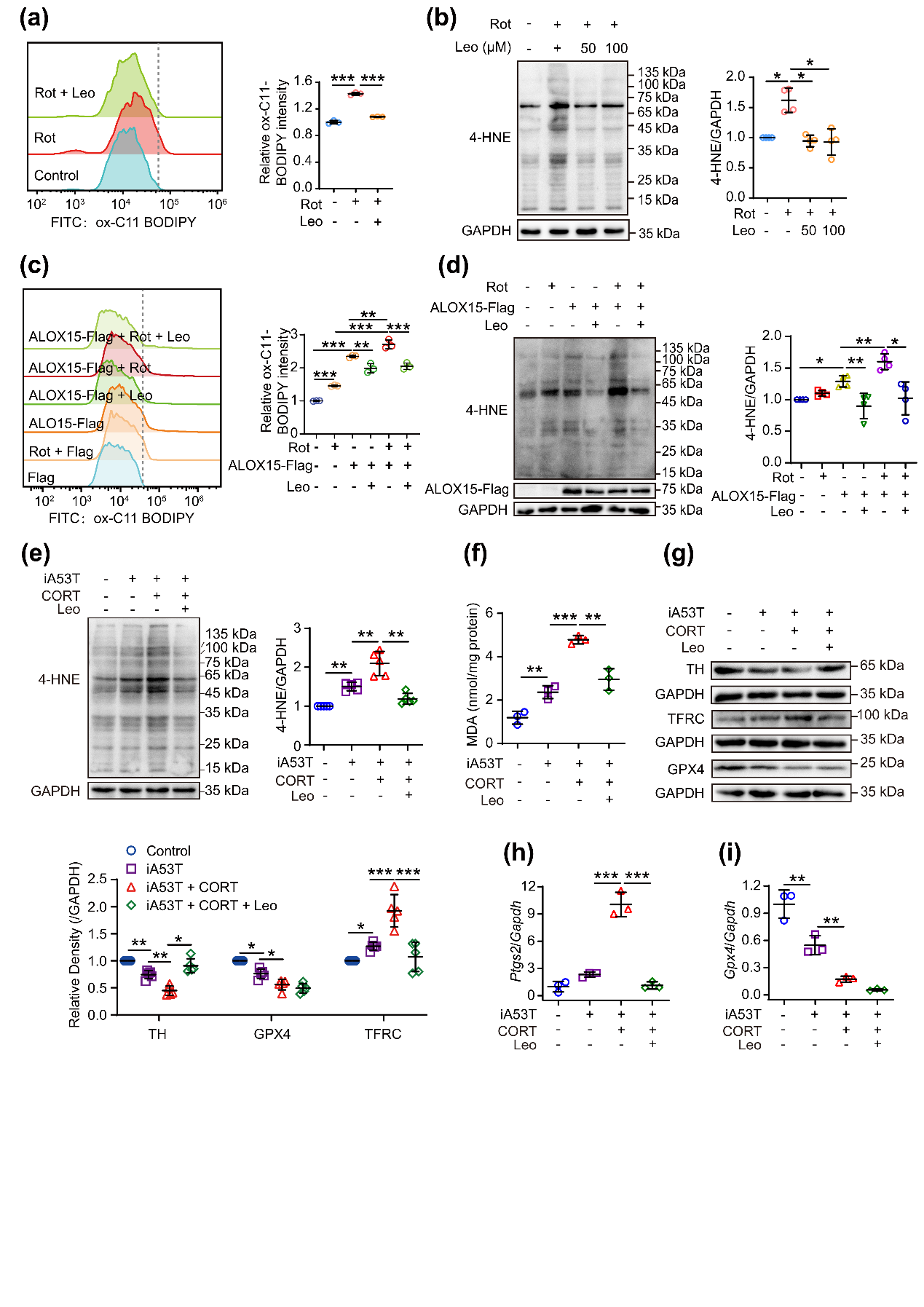


**Figure S6.** Leonurine protects cells via inhibiting lipid peroxidation. (a, b) Lipid peroxidation was detected by flow cytometry with C11 BODIPY (581/591) (*n* = 4) and western blot analysis of 4-HNE-protein adducts (*n* = 4) in PC12 cells treated with rotenone (0.75 μM) or leonurine (50 μM). (c, d) Lipid peroxidation was detected by flow cytometry with C11 BODIPY (581/591) (*n* = 3) and western blot analysis of 4-HNE-protein adducts (*n* = 4) in PC12 cells overexpressing ALOX15-Flag treated with rotenone (0.75 μM) or leonurine (50 μM). (e, g) The levels of 4-HNE-protein adducts and ferroptosis-related proteins were measured using western blot in *SNCA*-overexpressed PC12 cells treated with CORT (200 μM) or leonurine (50 μM) (*n* = 5). (f) The content of MDA was detected in PC12 cells (n = 3). (h, i) The change of *Ptgs2* and *Gpx4* mRNA expression were detected by RT-qPCR in PC12 cells (n = 3). All data represent mean ± SD. ^*^*p* < 0.05, ^**^*p* < 0.01 and ^***^*p* < 0.001, one-way ANOVA with Bonferroni test (for a, c, f, h, i), Dunnett T3 (for b), or LSD test (for d) or Tukey test (for e, g).


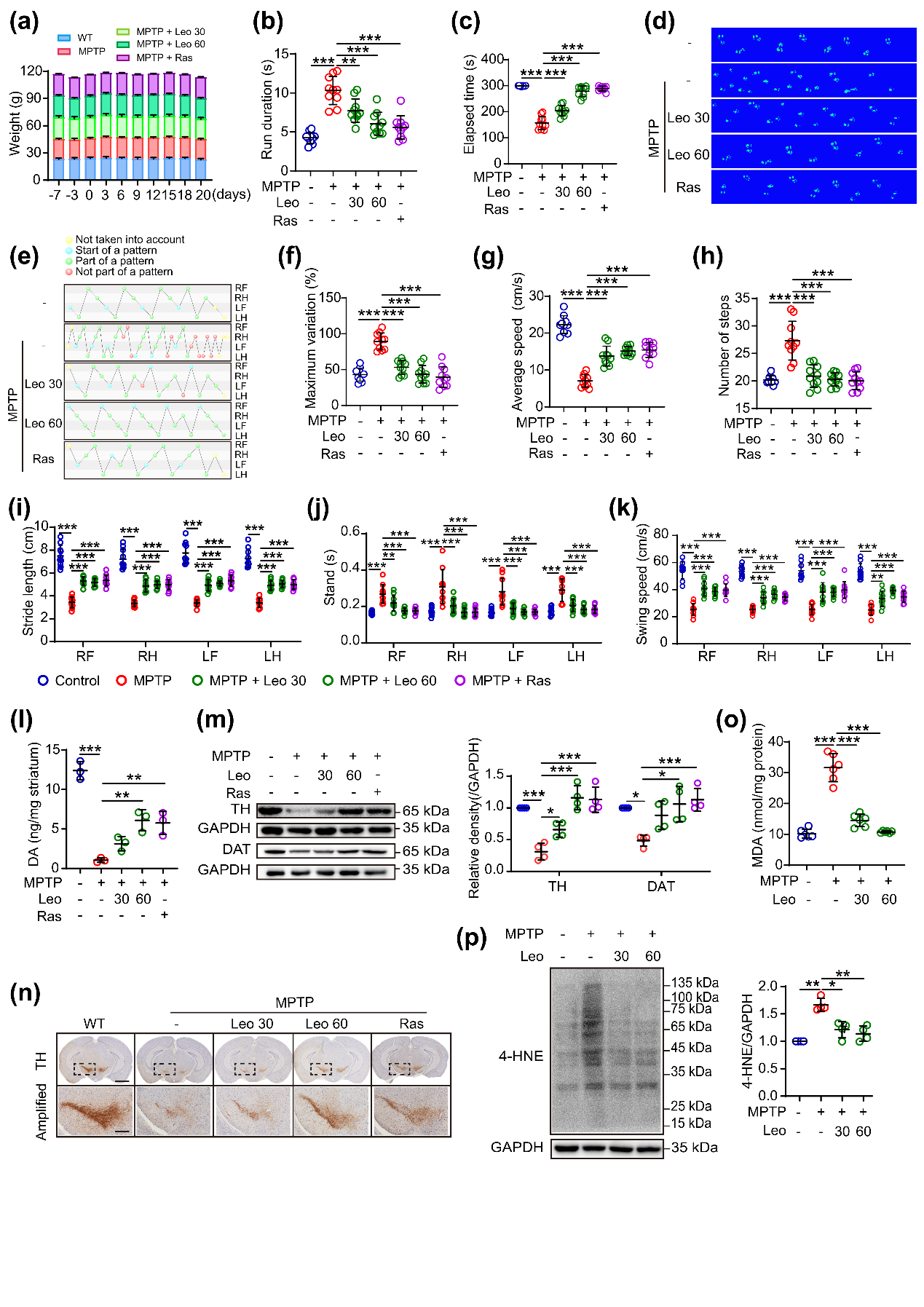


**Figure S7.** Leonurine alleviates behavioral disorder in MPTP-induced PD mouse model. (a) Percentage change in body weight of mice after treatment with MPTP (30 mg/kg for 5 days, *i.p.*) and leonurine (30 or 60 mg/kg/d, *i.g.*) or rasagiline (1 mg/kg/d, *i.g.*) (*n* = 10). (b-k) The pole test (b), rotarod test (c) and CatWalk gait analysis (d-k) were used to evaluate the motor function after MPTP and leonurine treatment in WT mice (*n* = 10). RF, right front. RH, right hind. LF, left front. LH, left hind. Green balls: normal pattern. Red balls: abnormal pattern. Blue balls: the beginning of a walking cycle. Yellow balls: data that were excluded from the test. (l) The dopamine was measured in striatum (*n* = 3). (m) Western blotting and quantitative analysis of TH expression in midbrain and DAT expression in striatum (*n* = 4). (n) IHC of coronal brain sections labeled with TH antibody and hematoxylin (upper, scale = 2 mm). The substantia nigra (dotted area) were amplified on bottom with scale = 500 μm (*n* = 3). (o) The content of MDA was measured in midbrain (*n* = 6). (p) Western blotting (left) and quantitative analysis (right) of 4-HNE-protein adducts in midbrain (*n* = 4). All data represent mean ± SD. ^*^*p* < 0.05, ^**^*p* < 0.01 and ^***^*p* < 0.001, by two-way ANOVA with Tukey (for a) and one-way ANOVA with Tukey (for b, c, f-k), Bonferroni (for l, m) or Dunnett T3 test (for o, p).


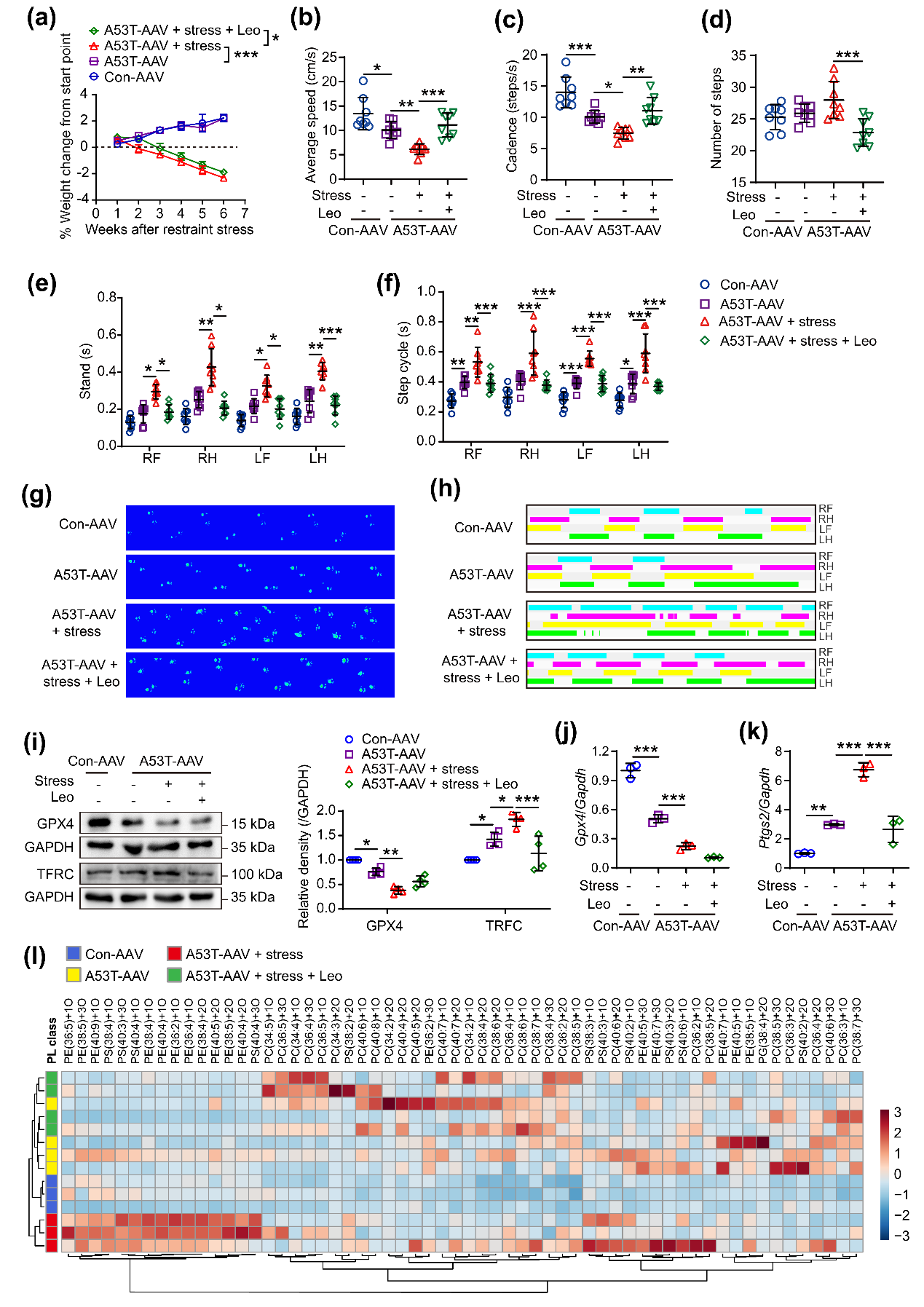


**Figure S8.** Leonurine mitigates stress-induced behavioral disorder in A53T-AAV PD mouse model. (a) Percentage change from start point in body weight of mice (*n* = 8). (b-f) Disordered motor coordination of mice was assessed by CatWalk gait analysis including average speed (b), cadence (c), number of steps (d), stand (e) and step cycle (f) (*n* = 8). (g, h) The gait pattern, referring to the order in which the mice complete a sequence of walking, was visualized as footprint view and footprint length (*n* = 8). RF, right front. RH, right hind. LF, left front. LH, left hind. (i) Western blotting (left) and quantitative analysis (right) of ferroptosis-related proteins in midbrain (n = 4). (j, k) The change of *Gpx4* and *Ptgs2* mRNA expressions in the midbrain were detected by RT-qPCR assay (n = 3). (l) Data of the oxidized phospholipids in the midbrain were extracted and displayed as heatmap. All data represent mean ± SD. ^*^*p* < 0.05, ^**^*p* < 0.01 and ^***^*p* < 0.001, by two-way ANOVA with Tukey (for a), one-way ANOVA with Tukey (for b-f), LSD (for i) or Bonferroni (for j, k).

**Table S1.** Primers sequences for RT-PCR

| **Gene** | **Forward Primer sequence (5’-3’)** | **Reverse Primer sequence (5’-3’)** |
| --- | --- | --- |
| ***Mus-Ptgs2*** | TGCACTATGGTTACAAAAGCTGG | TCAGGAAGCTCCTTATTTCCCTT |
| ***Mus-Gpx4*** | GCCTGGATAAGTACAGGGGTT | CATGCAGATCGACTAGCTGAG |
| ***Mus-Tfrc*** | ATGCCGACAATAACATGAAGGC | ACACGCTTACAATAGCCCAGG |
| ***Mus-Slc7a11*** | GGCACCGTCATCGGATCAG | CTCCACAGGCAGACCAGAAAA |
| ***Mus-Acsl4*** | TCCTCCAAGTAGACCAACCCC | AGTCCAGGGATACGTTCACAC |
| ***Mus-Lpcat3*** | CTACCCGTTGGCTCTGTTTTAC | TGAAGCACGACACATAGCAAG |
| ***Mus-Ncoa4*** | TGGTTGGTGACTCCTCAGGAA | TCACTCACATTGTAGGGCTC |
| ***Mus-Dmt1*** | TACCTAGACCCAGGAAACATCG | CACTCCAAGTCTCGCTGCAA |
| ***Mus-Gapdh*** | AAGAAGGTGGTGAAGCAGG | GAAGGTGGAAGAGTGGGAGT |
|  |  |  |
| ***Rat-Slc7a11*** | ATCGGATCGGGCATCTTCAT | GGGACCAAAGACCTCCAGAA |
| ***Rat-Gpx4*** | CCAGACTCATTCAACCAGACA | GATGACTGAGTACCTGAACCG |
| ***Rat-Pla2g6*** | AACTCAGCCTCCAACGATCA | GTGGTCATGGCTCTCAGACT |
| ***Rat-Tfrc*** | GATCGGCTACCTGGGCTATT | TGTATTCTGGCTCAGCTGCT |
| ***Rat-Acsl4*** | CCATATCGCTCTGTCACGCACTT | CCCCAGGCTGTCCTTCTTCC |
| ***Rat-Alox15*** | GCTGAAGCGCTCTACTTGTC | GGAAGTTCAAGCTGGATGGC |
| ***Rat-Ptgs2*** | AACTCCAGTGCCTGAGACCA | TGGTCTCCCCAAAGATAGCA |

**Table S2.** The docking parameters of TG compounds

| **Index** | **Name** | **-CDOCKER_ENERGY** | **-**  **CDOCKER_INTERACTION** |
| --- | --- | --- | --- |
| 1 | Leonurine | 30.4162 | 35.7575 |
| 2 | Cianidanol | 23.3628 | 29.6417 |
| 3 | Epicatechin | 22.8702 | 28.8622 |
| 4 | L-Phenylalanine | 17.5097 | 18.1602 |
| 5 | Wogonin | 16.955 | 25.6267 |
| 6 | Baicalin | 9.04608 | 38.2129 |
| 7 | Wogonoside | 7.41221 | 45.3487 |
| 8 | Baicalein 6-O-glucoside | 5.43339 | 36.7178 |
| 9 | Oroxylin A glucoronide | 1.83326 | 36.7727 |
| 10 | 2,3,4',5-Tetrahydroxystilbene 2-o-D-glucoside | -1.47291 | 31.8917 |
| 11 | Rhynchophylline | -31.4449 | 31.6683 |
| 12 | Geniposide | -32.9838 | 34.8162 |
| 13 | Isorhynchophylline | -33.2444 | 28.1515 |
| 14 | Isocorynoxeine | -37.5211 | 29.291 |
| 15 | corynoxeine | -40.6256 | 29.8946 |
| 16 | Genipin 1-gentiobioside | -46.5227 | 44.5958 |
| 17 | cyasteron | -53.7319 | 31.3351 |

**Table S3.** List of AAVs and plasmids

| **Category** | **Company** |
| --- | --- |
| AAV2/9-hEF1a-DIO-EGFP-WPRE-pA | Taitool Bioscience (Shanghai,  China) |
| AAV2/9-hEF1a-DIO-EGFP-P2A-SNCA(A53T)-WPRE-pA | Taitool Bioscience (Shanghai,  China) |
| pCDNA3.1-ALOX15-EGFP | Transheep (Shanghai, China) |
| pCDNA3.1-PEBP1-HA | Transheep (Shanghai, China) |
| pCDNA3.1-ALOX15-3×Flag | Transheep (Shanghai, China) |
